# Supplementary material for: The effect of crystallite size on pressure amplification in switchable porous solids
Source: Nat Commun. 2018 Apr 20;9:1573. doi: 10.1038/s41467-018-03979-2 (PMC5910435; doi:10.1038/s41467-018-03979-2)
Supplement: Supplementary file 1 — Supplementary Information [file 41467_2018_3979_MOESM1_ESM.pdf]

Supplementary Information:

The effect of crystallite size on pressure amplification in switchable porous solids

Krause *et al.*

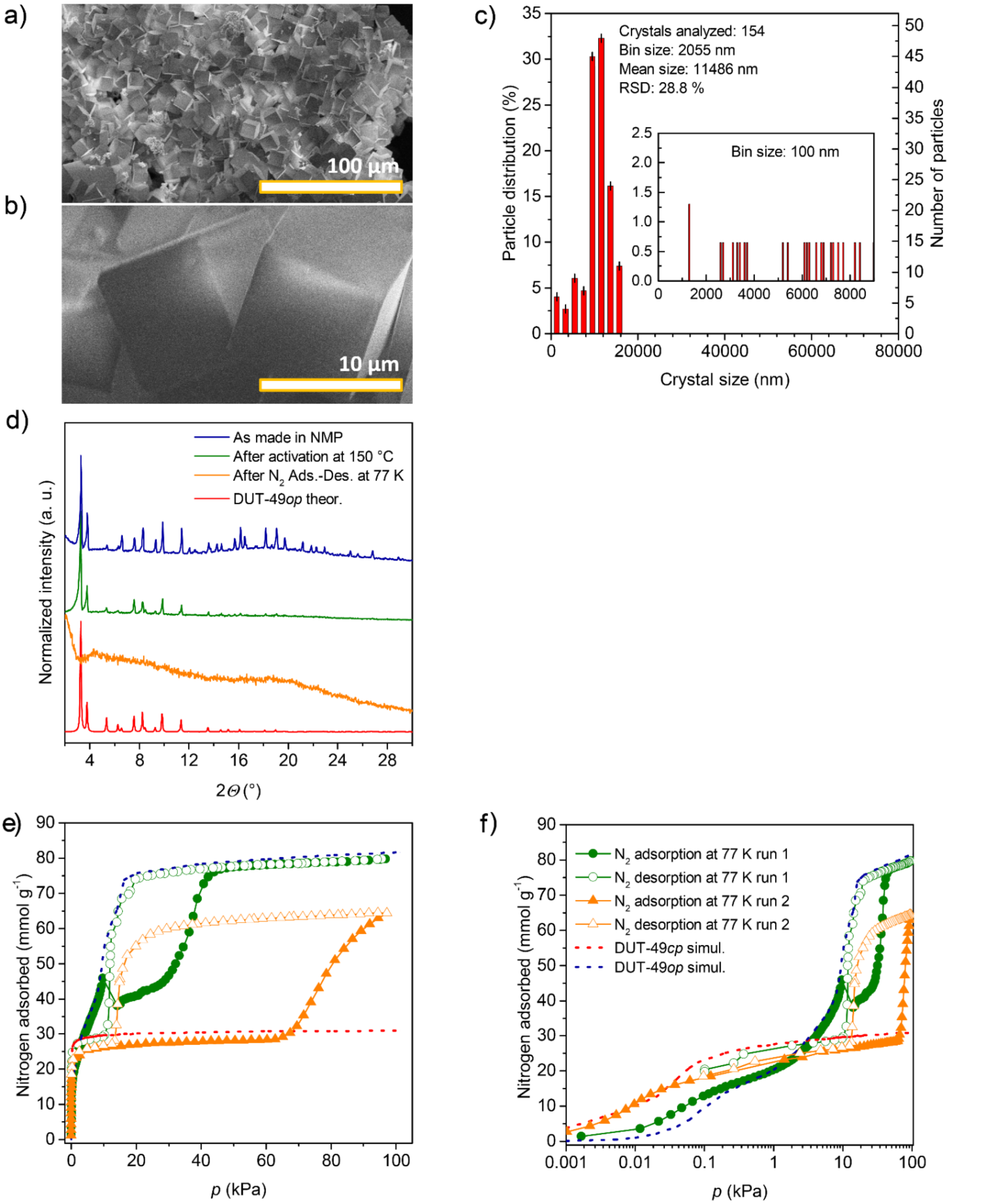

Supplementary Figure 1. Characterization of DUT-49(**1**) (mean size: 11.4 µm): SEM images (a, b); crystal size distribution derived from SEM images (inlet: magnification of area with smaller crystal sizes and smaller bin size) (c); PXRD patters of: as made (blue), activated (green), after N_2 adsorption-desorption at 77 K (orange) and theoretical DUT-49*op* (d); N_2 physisorption isotherms at 77 K measured in two consecutive cycles (simulated isotherms for DUT-49*op* (blue dashed line) and DUT-49*cp* (red dashed line) are also shown) (e); N_2 physisorption isotherms at 77 K as semi-logarithmic plot (f).

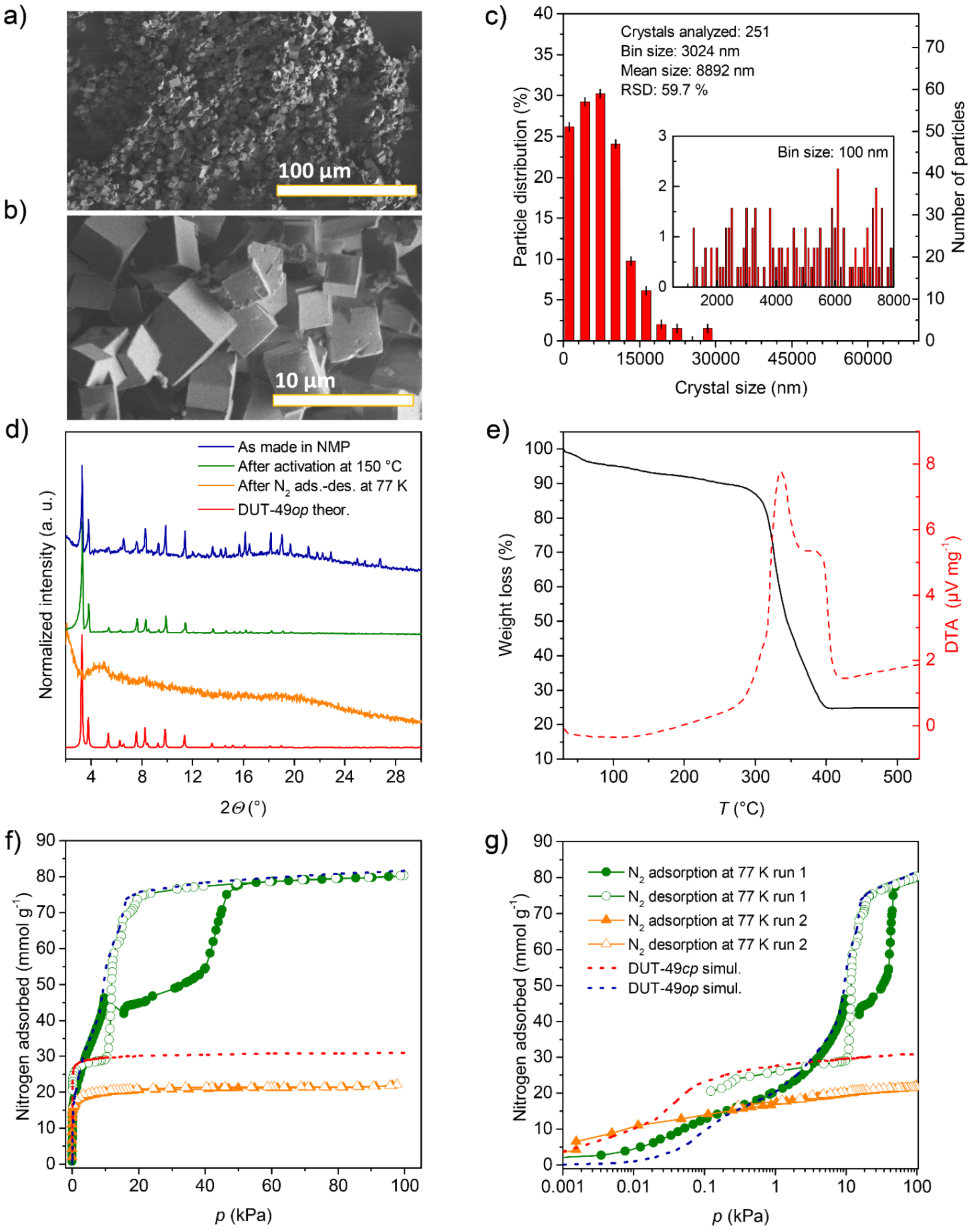

Supplementary Figure 2. Characterization of DUT-49(**2**) (mean size: 8.89 µm): SEM images (a, b); crystal size distribution derived from SEM images, inlet: magnification of area with smaller crystal sizes and smaller bin size (c); PXRD patters of as made (blue), activated (green), after N_2 adsorption-desorption at 77 K (orange) and theoretical DUT-49*op* (d); TGA (e); N_2 physisorption at 77 K measured in two consecutive cycles (including simulated isotherms for DUT-49*op* (blue dashed line) and DUT-49*cp* (red dashed line) (f); N_2 physisorption isotherms at 77 K as semi-logarithmic plot (g).

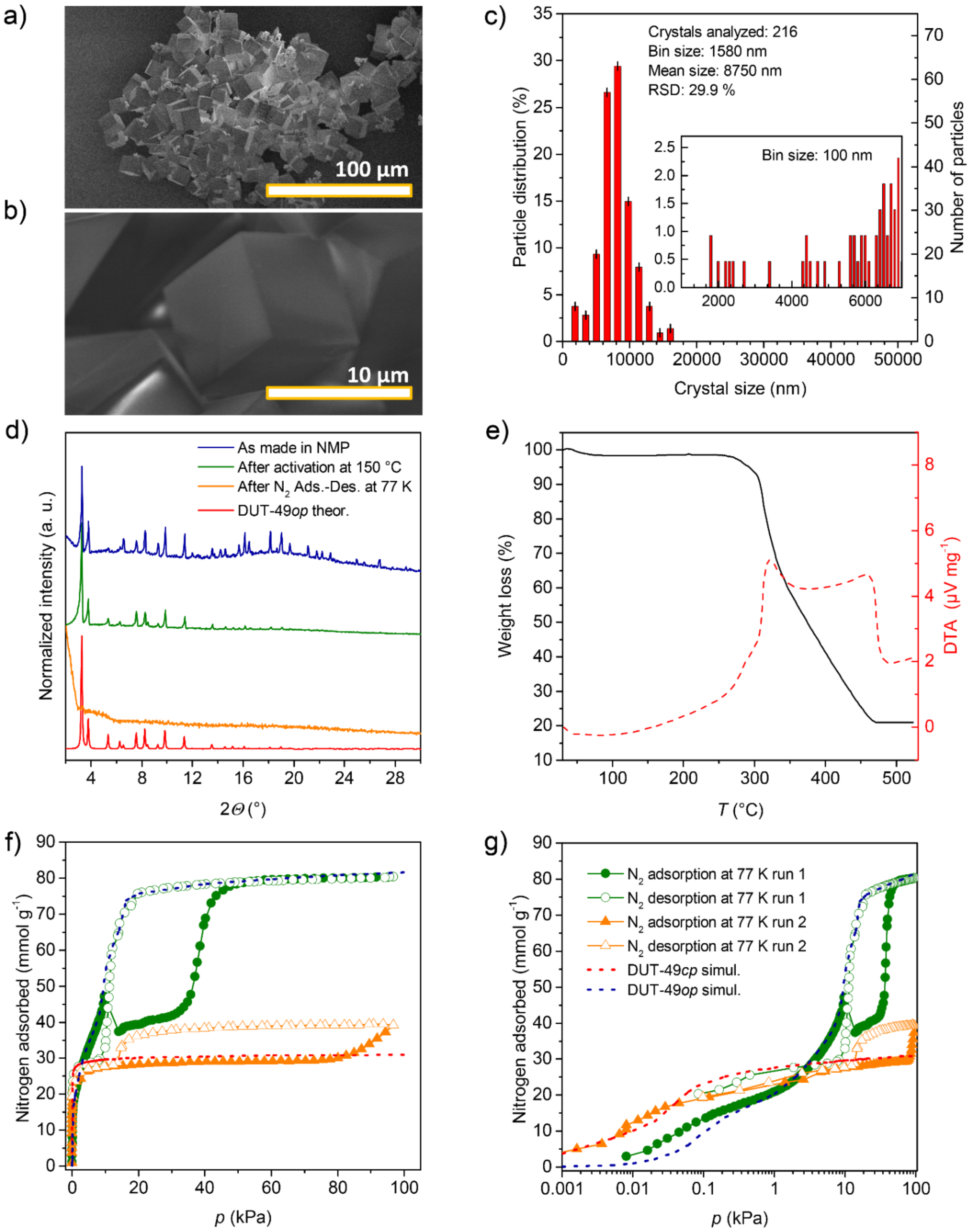

Supplementary Figure 3. Characterization of DUT-49(**3**) (mean size: 8.75 µm): SEM images (a, b); crystal size distribution derived from SEM images, inlet: magnification of area with smaller crystal sizes and smaller bin size (c); PXRD patters of as made (blue), activated (green), after N_2 adsorption-desorption at 77 K (orange) and theoretical DUT-49*op* (d); TGA (e); and N_2 physisorption at 77 K measured for two consecutive cycles including simulated isotherms for DUT-49*op* (blue dashed line) and DUT-49*cp* (red dashed line) (f); N_2 physisorption isotherms at 77 K as semi-logarithmic plot (g).

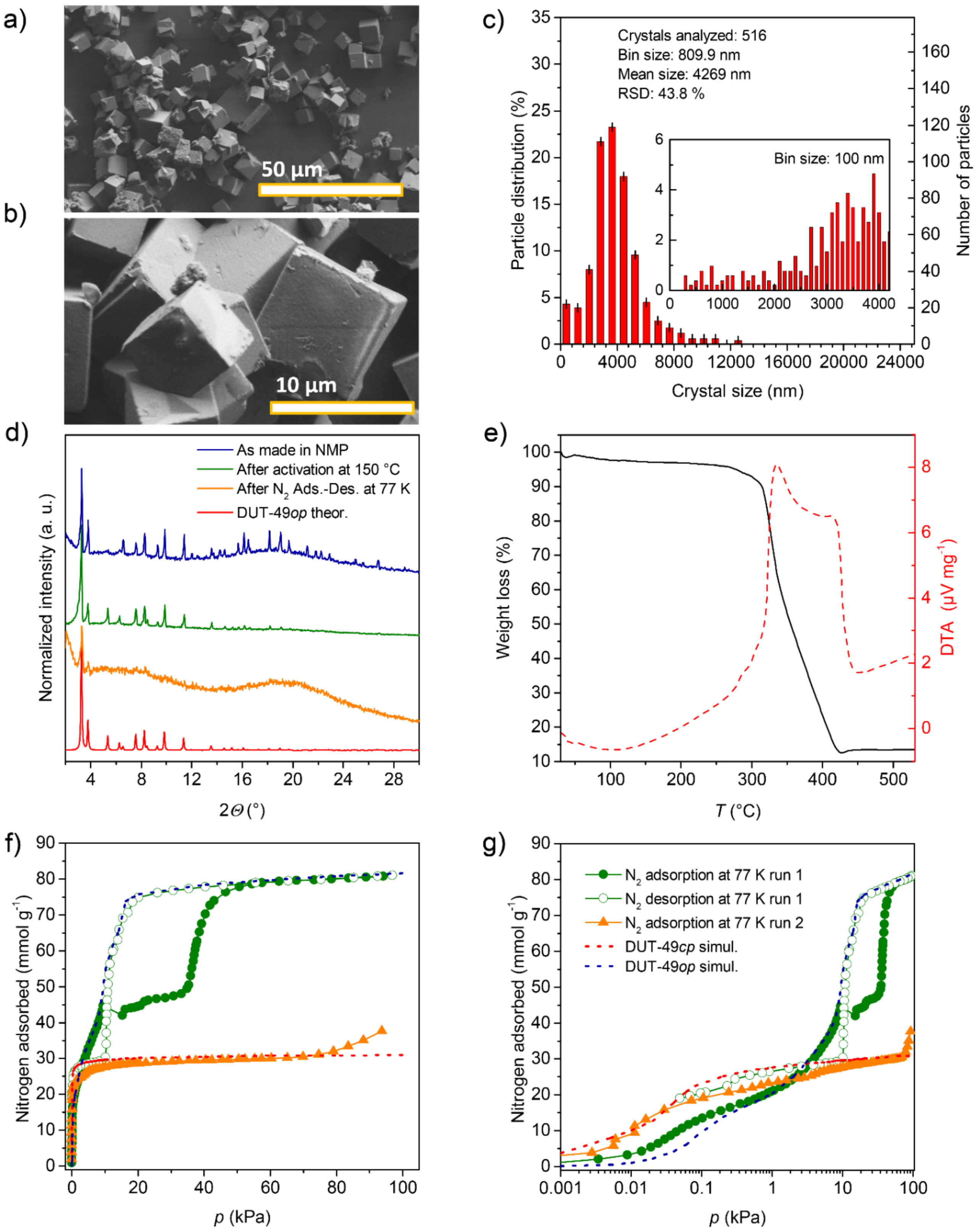

Supplementary Figure 4. Characterization of DUT-49(**4**) (mean size: 4.27 µm): SEM images (a, b); crystal size distribution derived from SEM images, inlet: magnification of area with smaller crystal sizes and smaller bin size (c); PXRD patters of as made (blue), activated (green), after N_2 adsorption-desorption at 77 K (orange) and theoretical DUT-49*op* (d); TGA (e); and N_2 physisorption at 77 K measured for two consecutive cycles including simulated isotherms for DUT-49*op* (blue dashed line) and DUT-49*cp* (red dashed line) (f); N_2 physisorption isotherms at 77 K as semi-logarithmic plot (g).

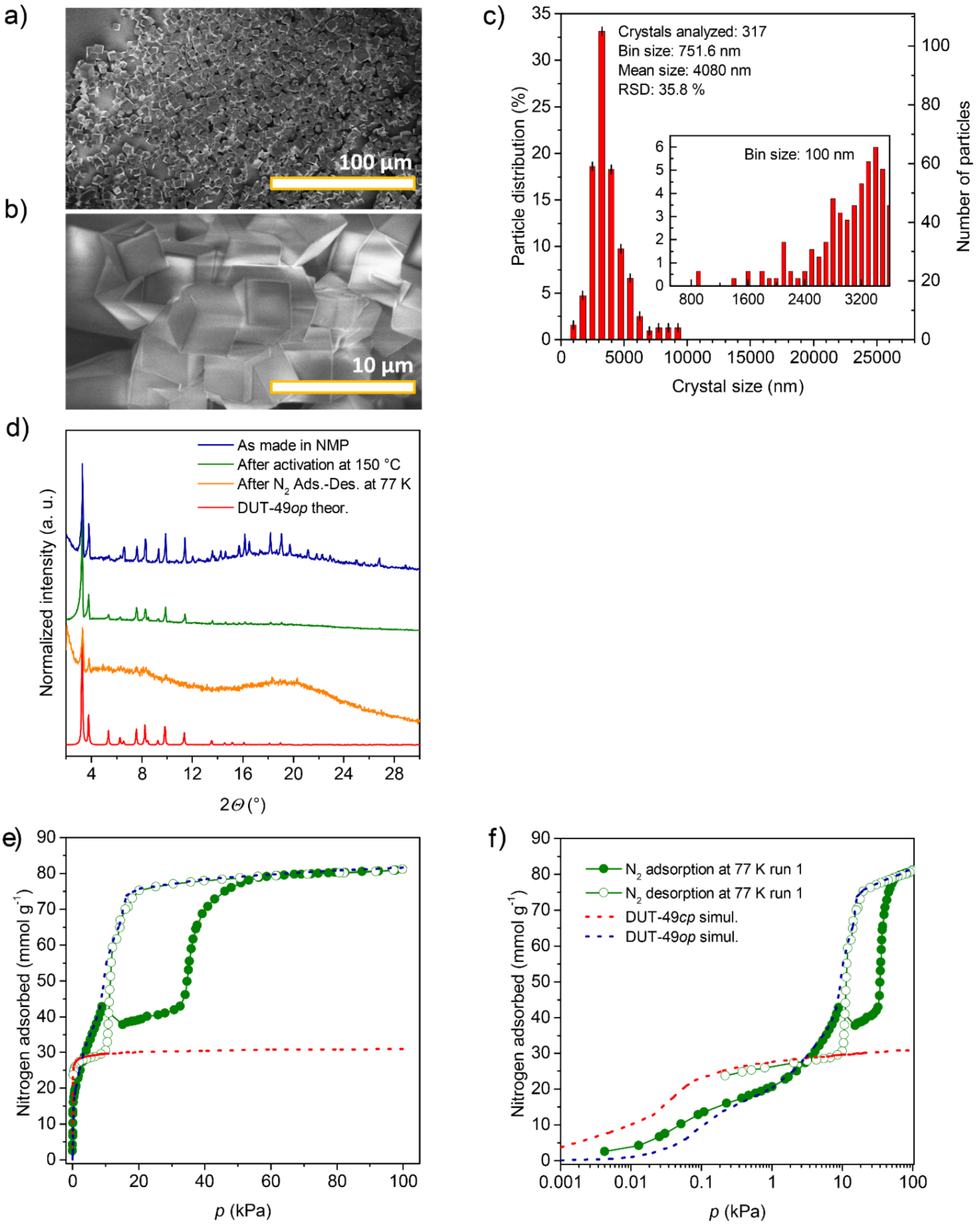

Supplementary Figure 5. Characterization of DUT-49(**5**) (mean size: 4.08 µm): SEM images (a, b); crystal size distribution derived from SEM images, inlet: magnification of area with smaller crystal sizes and smaller bin size (c); PXRD patters of as made (blue), activated (green), after N_2 adsorption-desorption at 77 K (orange) and theoretical DUT-49*op* (d); N_2 physisorption at 77 K including simulated isotherms for DUT-49*op* (blue dashed line) and DUT-49*cp* (red dashed line) (e); N_2 physisorption isotherms at 77 K as semi-logarithmic plot (f).

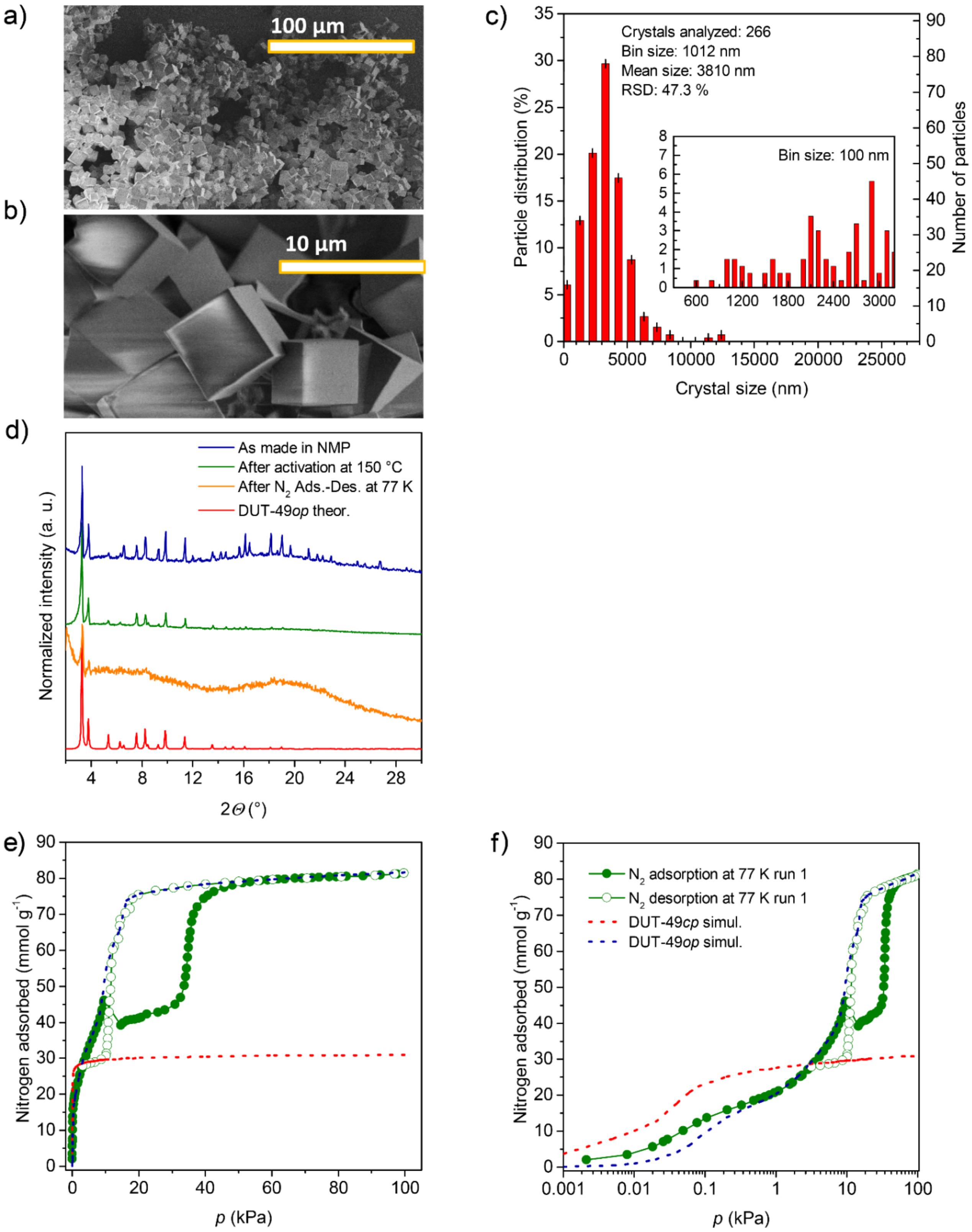

Supplementary Figure 6. Characterization of DUT-49(**6**) (mean size: 3.81 µm): SEM images (a, b); crystal size distribution derived from SEM images, inlet: magnification of area with smaller crystal sizes and smaller bin size (c); PXRD patters of as made (blue), activated (green), after N_2 adsorption-desorption at 77 K (orange) and theoretical DUT-49*op* (d); N_2 physisorption at 77 K including simulated isotherms for DUT-49*op* (blue dashed line) and DUT-49*cp* (red dashed line) (e); N_2 physisorption isotherms at 77 K as semi-logarithmic plot (f).

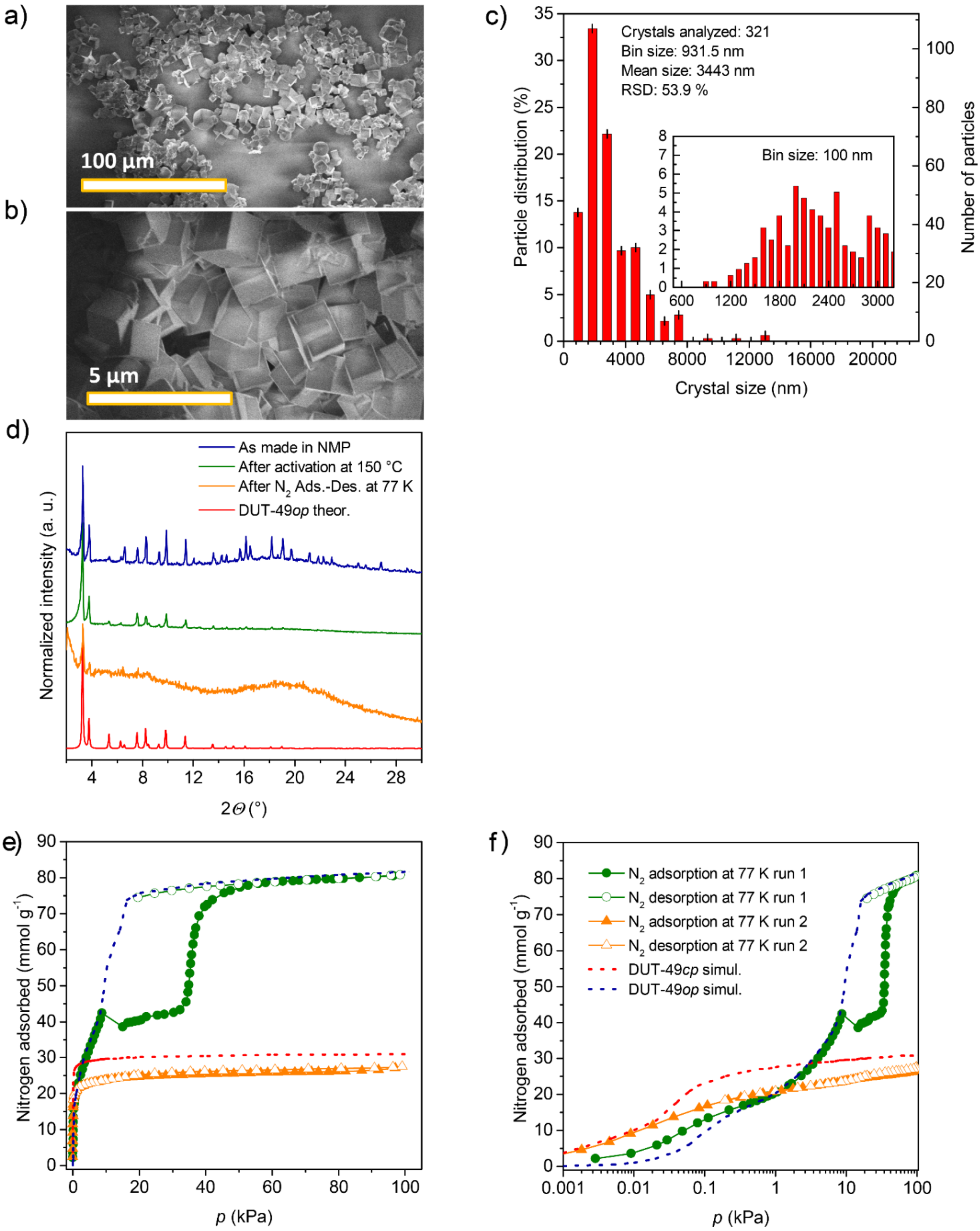

Supplementary Figure 7. Characterization of DUT-49(**7**) (mean size: 3.44 µm): SEM images (a, b); crystal size distribution derived from SEM images, inlet: magnification of area with smaller crystal sizes and smaller bin size (c); PXRD patterns of as made (blue), activated (green), after N_2 adsorption-desorption at 77 K (orange) and theoretical DUT-49*op* (d); N_2 physisorption at 77 K measured for two consecutive cycles including simulated isotherms for DUT-49*op* (blue dashed line) and DUT-49*cp* (red dashed line) (e); N_2 physisorption isotherms at 77 K as semi-logarithmic plot (f).

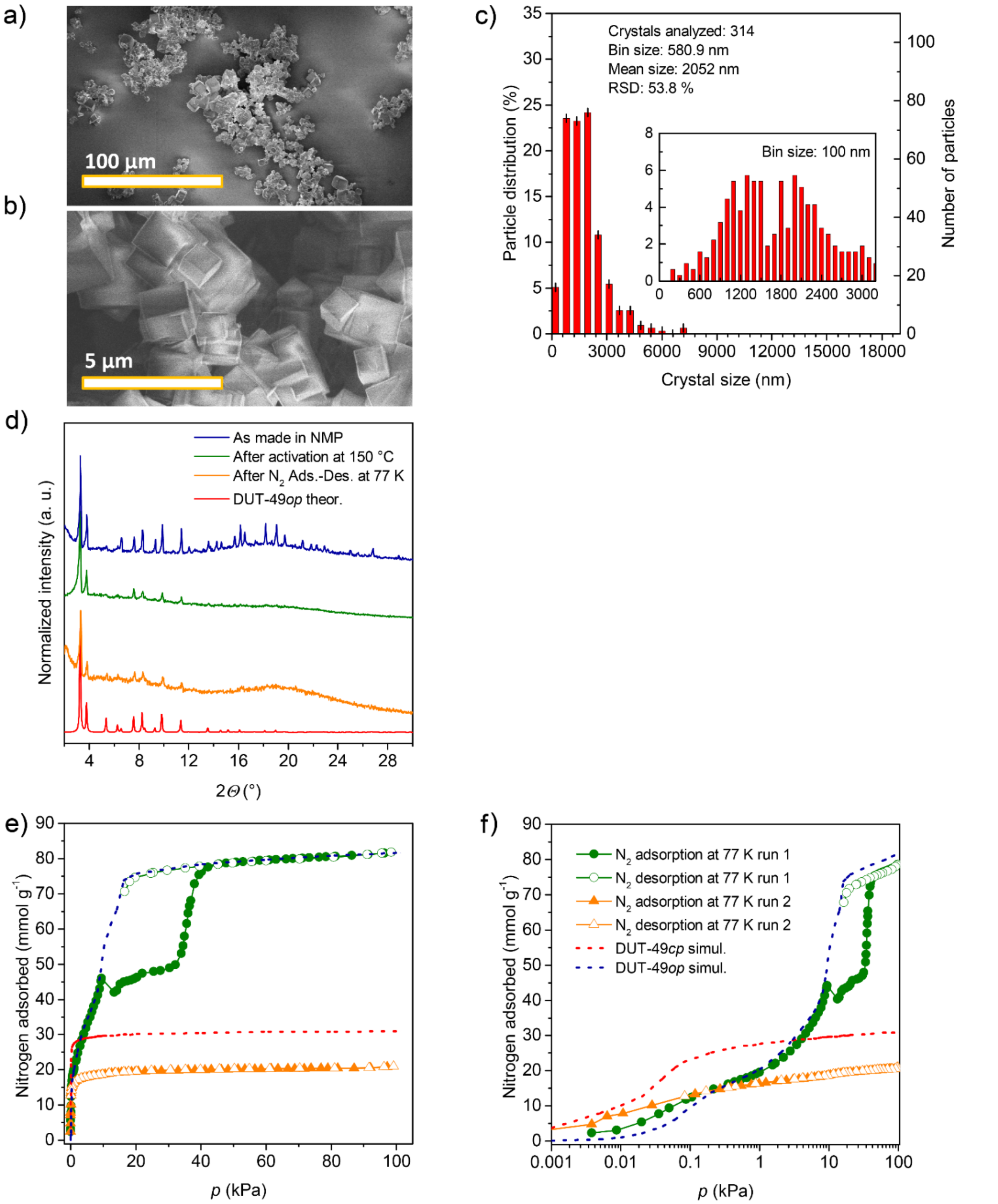

Supplementary Figure 8. Characterization of DUT-49(**8**) (mean size: 2.05 µm): SEM images (a, b); crystal size distribution derived from SEM images, inlet: magnification of area with smaller crystal sizes and smaller bin size (c); PXRD patters of as made (blue), activated (green), after N_2 adsorption-desorption at 77 K (orange) and theoretical DUT-49*op* (d); and N_2 physisorption at 77 K measured for two consecutive cycles including simulated isotherms for DUT-49*op* (blue dashed line) and DUT-49*cp* (red dashed line) (e); N_2 physisorption isotherms at 77 K as semi-logarithmic plot (f).

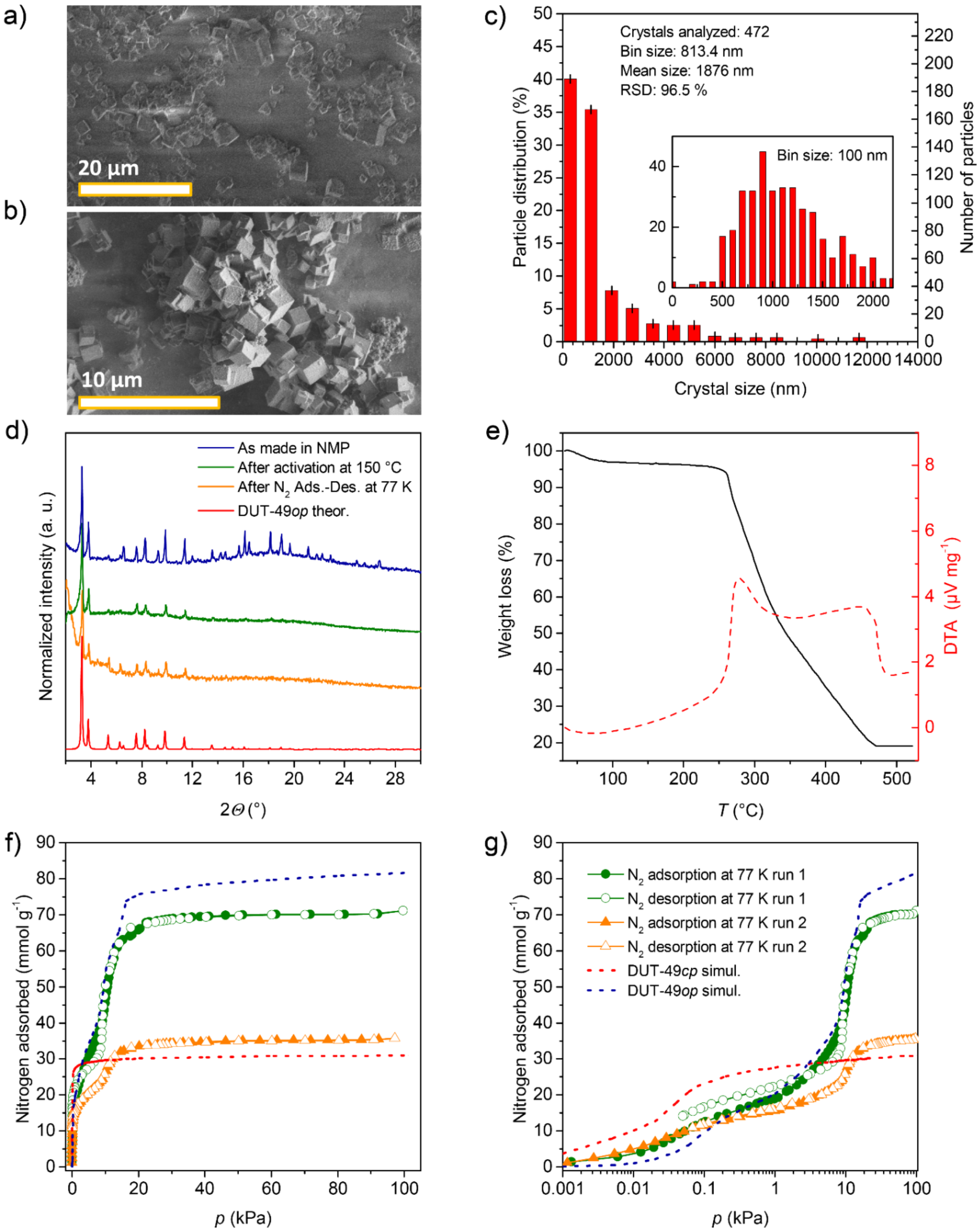

Supplementary Figure 9. Characterization of DUT-49(**9**) (mean size: 1.88 µm): SEM images (a, b); crystal size distribution derived from SEM images, inlet: magnification of area with smaller crystal sizes and smaller bin size (c); PXRD patters of as made (blue), activated (green), after N_2 adsorption-desorption at 77 K (orange) and theoretical DUT-49*op* (d); TGA (e); and N_2 physisorption at 77 K measured for two consecutive cycles including simulated isotherms for DUT-49*op* (blue dashed line) and DUT-49*cp* (red dashed line) (f); N_2 physisorption isotherms at 77 K as semi-logarithmic plot (g).

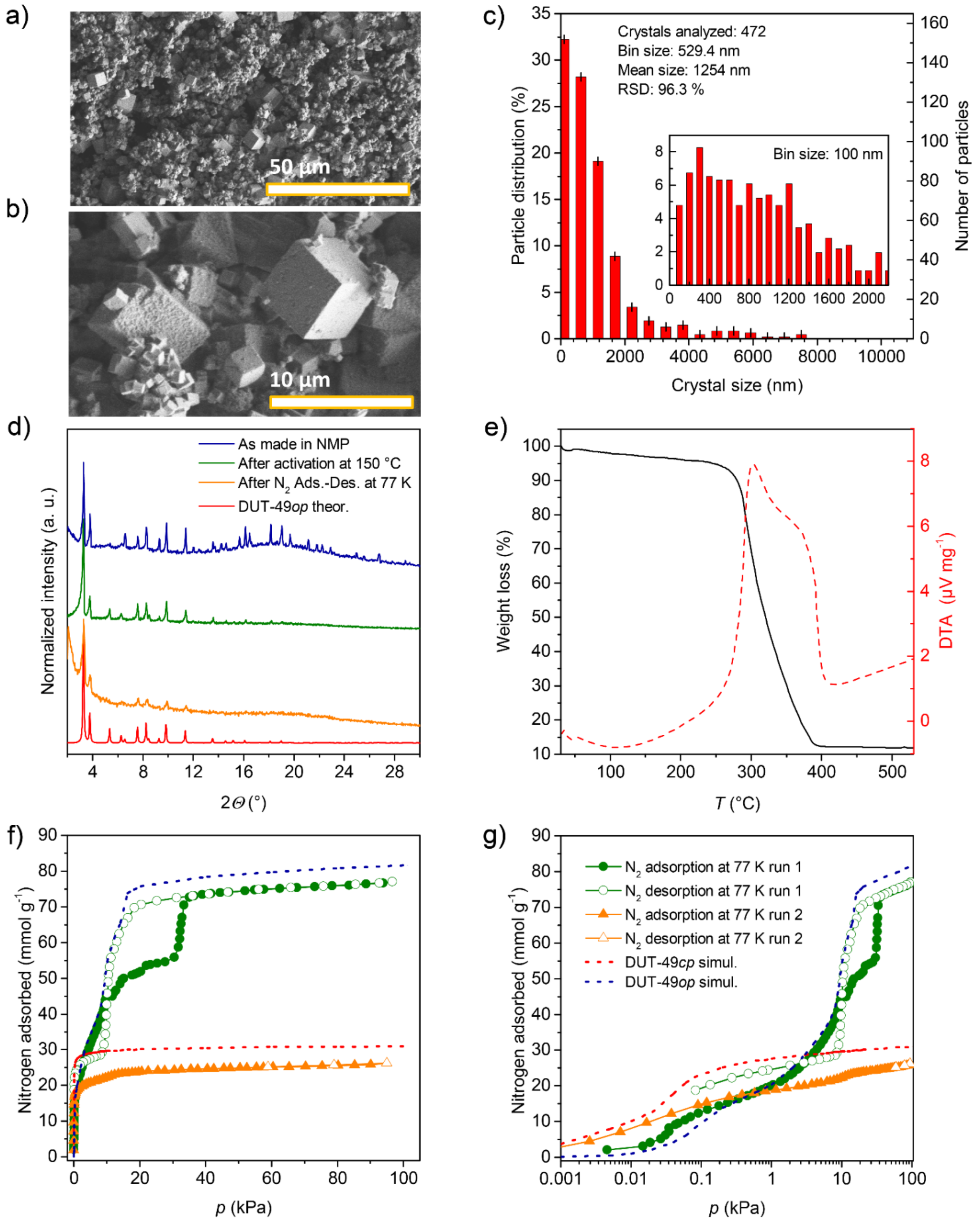

Supplementary Figure 10. Characterization of DUT-49(**10**) (mean size: 1.25 µm): SEM images (a, b); crystal size distribution derived from SEM images, inlet: magnification of area with smaller crystal sizes and smaller bin size (c); PXRD patterns of as made (blue), activated (green), after N_2 adsorption-desorption at 77 K (orange) and theoretical DUT-49*op* (d); TGA (e); and N_2 physisorption at 77 K measured for two consecutive cycles including simulated isotherms for DUT-49*op* (blue dashed line) and DUT-49*cp* (red dashed line) (f); N_2 physisorption isotherms at 77 K as semi-logarithmic plot (g).

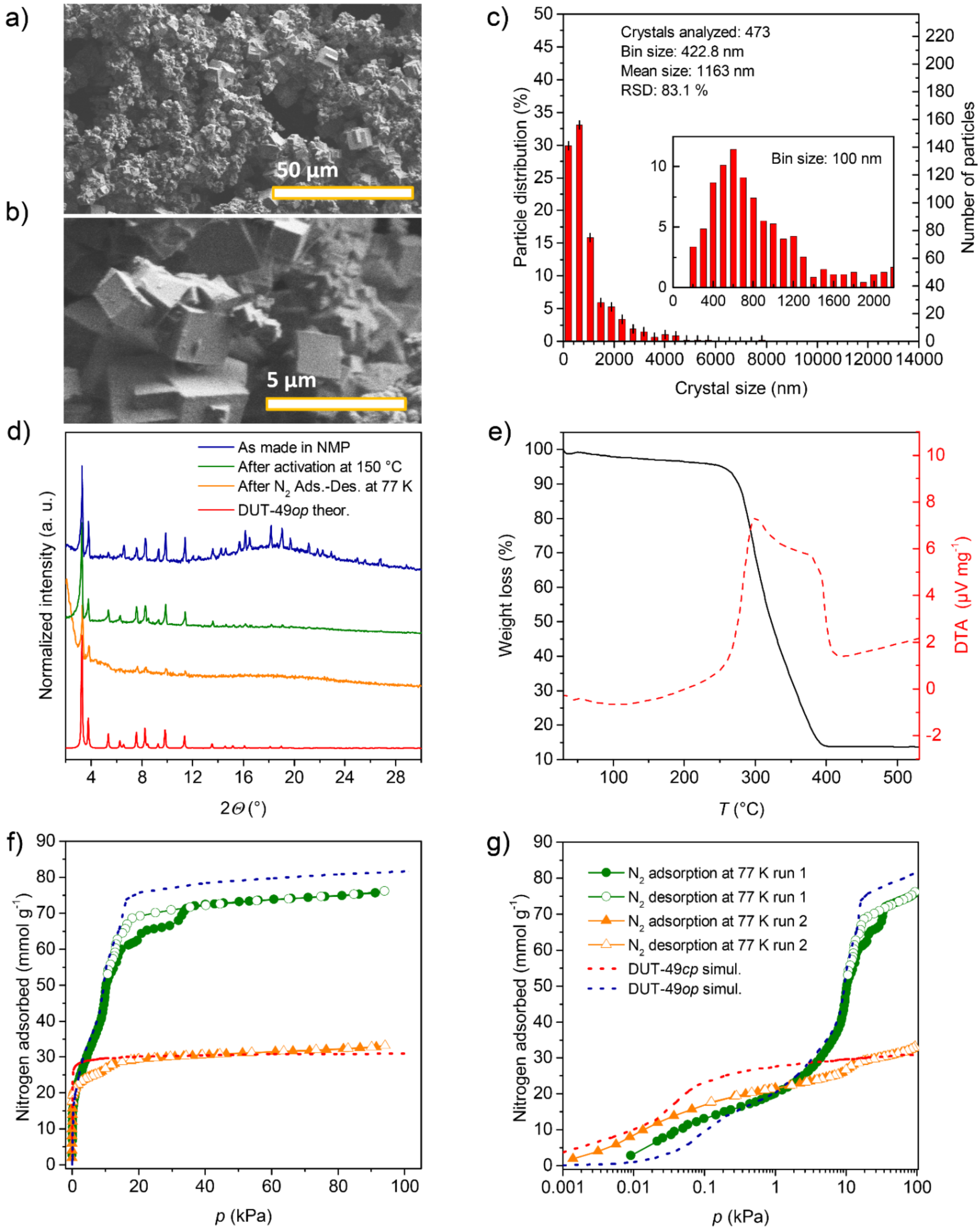

Supplementary Figure 11. Characterization of DUT-49(**11**) (mean size: 1.16 µm): SEM images (a, b); crystal size distribution derived from SEM images, inlet: magnification of area with smaller crystal sizes and smaller bin size (c); PXRD patterns of as made (blue), activated (green), after N_2 adsorption-desorption at 77 K (orange) and theoretical DUT-49*op* (d); TGA (e); and N_2 physisorption at 77 K measured for two consecutive cycles including simulated isotherms for DUT-49*op* (blue dashed line) and DUT-49*cp* (red dashed line) (f); N_2 physisorption isotherms at 77 K as semi-logarithmic plot (g).

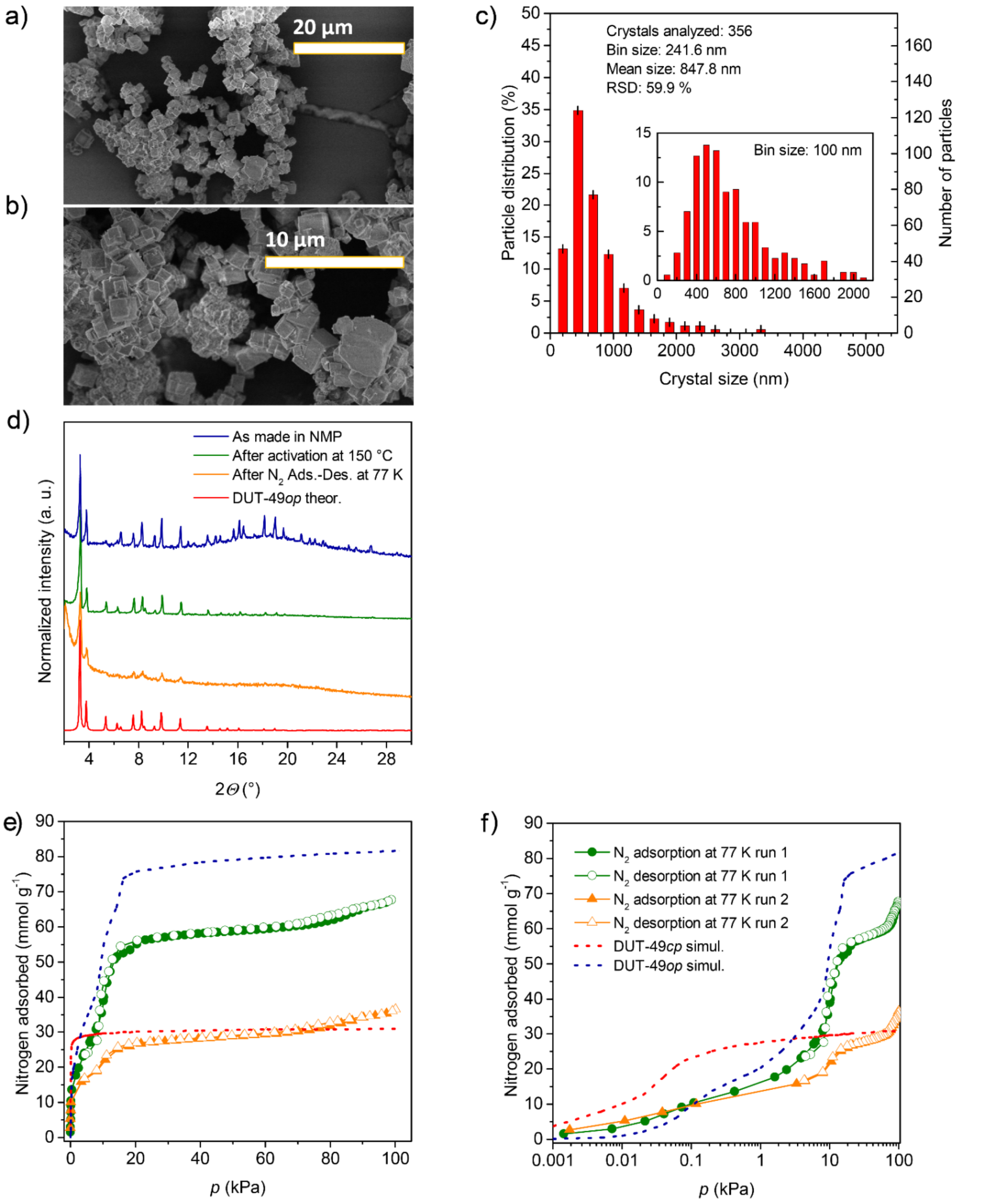

Supplementary Figure 12. Characterization of DUT-49(**12**) (mean size: 848 nm): SEM images (a, b); crystal size distribution derived from SEM images, inlet: magnification of area with smaller crystal sizes and smaller bin size (c); PXRD patters of as made (blue), activated (green), after N_2 adsorption-desorption at 77 K (orange) and theoretical DUT-49*op* (d); and N_2 physisorption at 77 K measured for two consecutive cycles including simulated isotherms for DUT-49*op* (blue dashed line) and DUT-49*cp* (red dashed line) (e); N_2 physisorption isotherms at 77 K as semi-logarithmic plot (f).

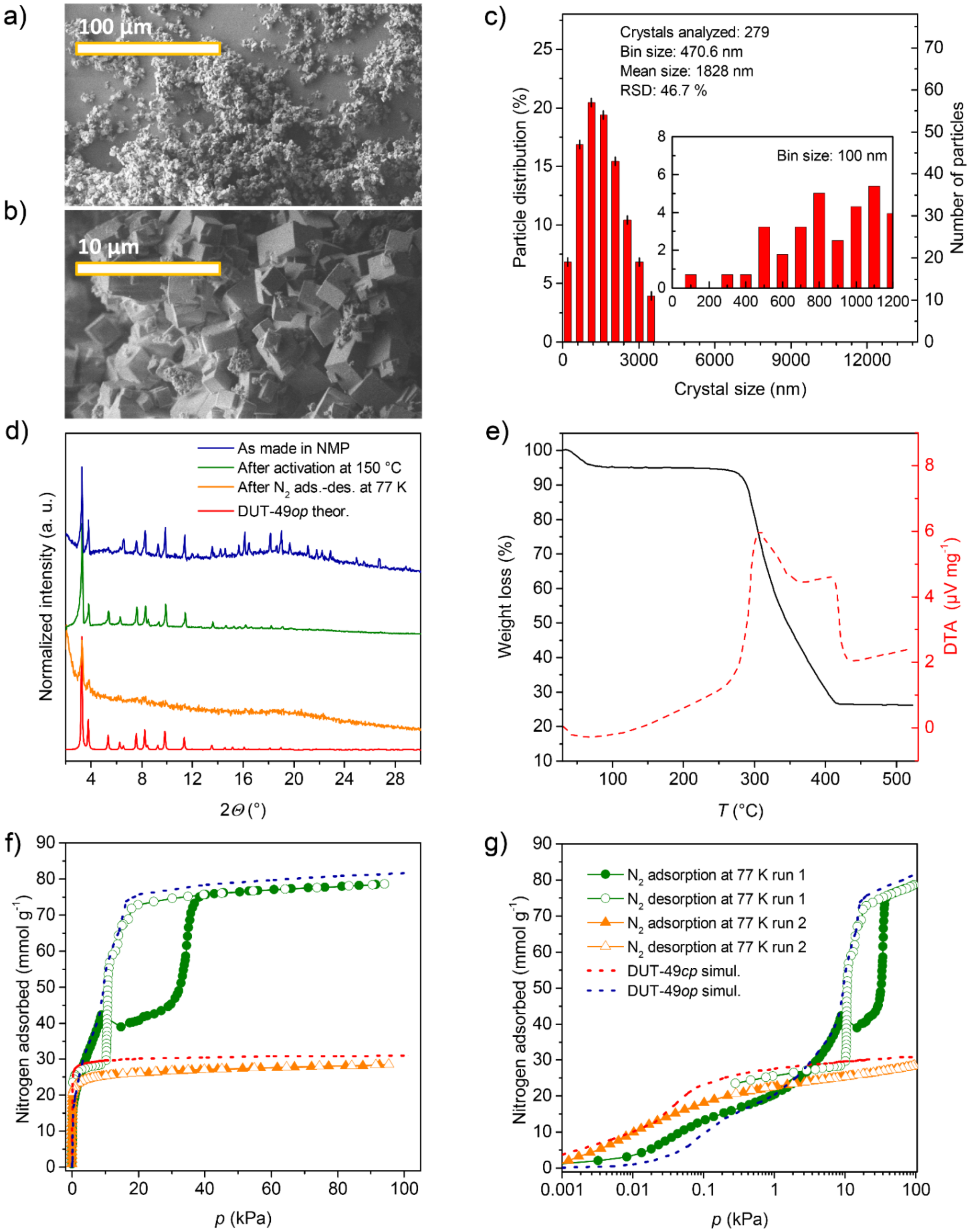

Supplementary Figure 13. Characterization of DUT-49(**13**) (mean size: 1.83 µm): SEM images (a, b); crystal size distribution derived from SEM images, inlet: magnification of area with smaller crystal sizes and smaller bin size (c); PXRD patters of as made (blue), activated (green), after N_2 adsorption-desorption at 77 K (orange) and theoretical DUT-49*op* (d); TGA (e); and N_2 physisorption at 77 K measured for two consecutive cycles including simulated isotherms for DUT-49*op* (blue dashed line) and DUT-49*cp* (red dashed line) (f); N_2 physisorption isotherms at 77 K as semi-logarithmic plot (g).

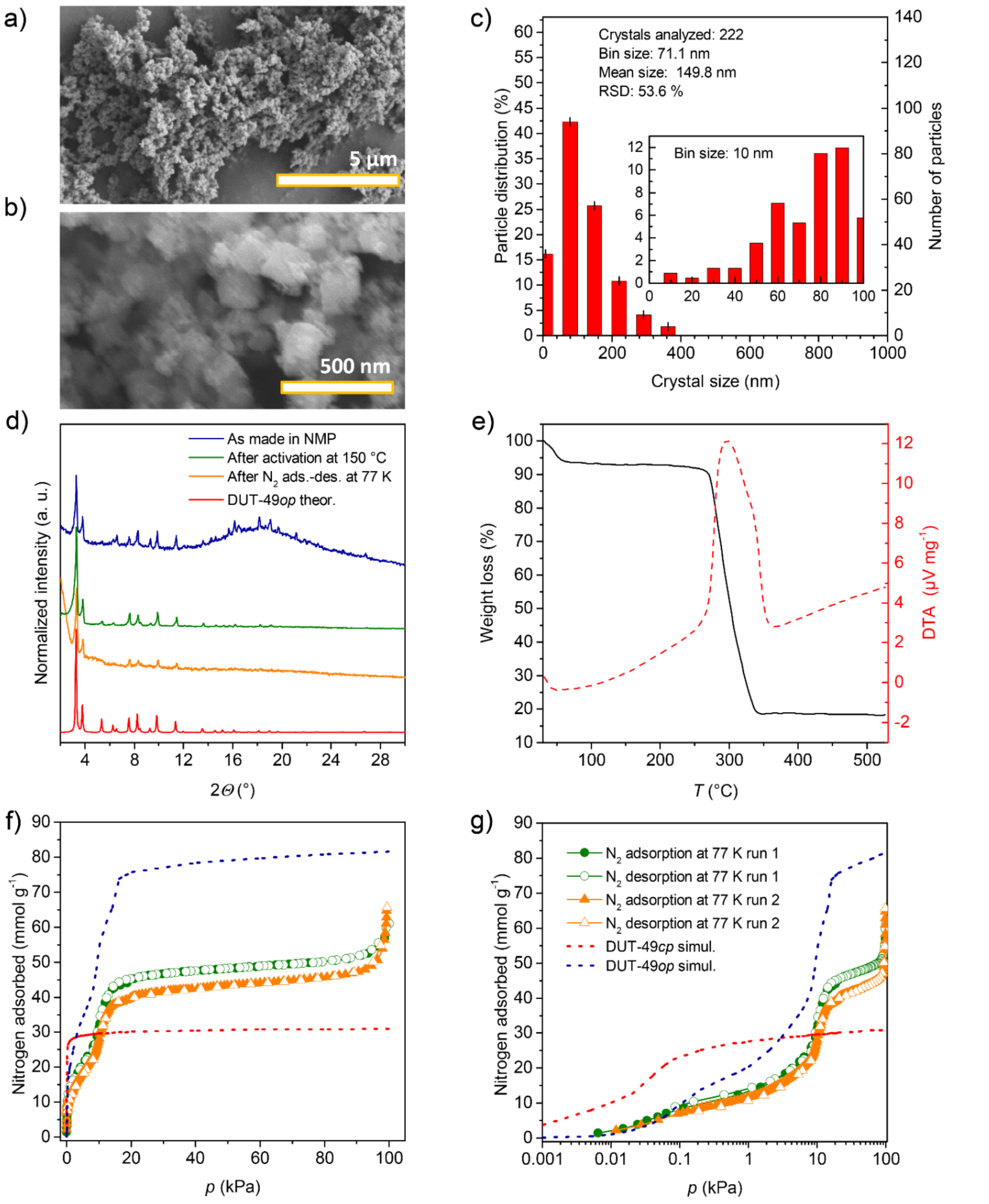

Supplementary Figure 14. Characterization of DUT-49(**14**) (mean size: 150 nm): SEM images (a, b); crystal size distribution derived from SEM images, inlet: magnification of area with smaller crystal sizes and smaller bin size (c); PXRD patterns of as made (blue), activated (green), after N_2 adsorption-desorption at 77 K (orange) and theoretical DUT-49*op* (d); TGA (e); and N_2 physisorption at 77 K measured for two consecutive cycles including simulated isotherms for DUT-49*op* (blue dashed line) and DUT-49*cp* (red dashed line) (f); N_2 physisorption isotherms at 77 K as semi-logarithmic plot (g).

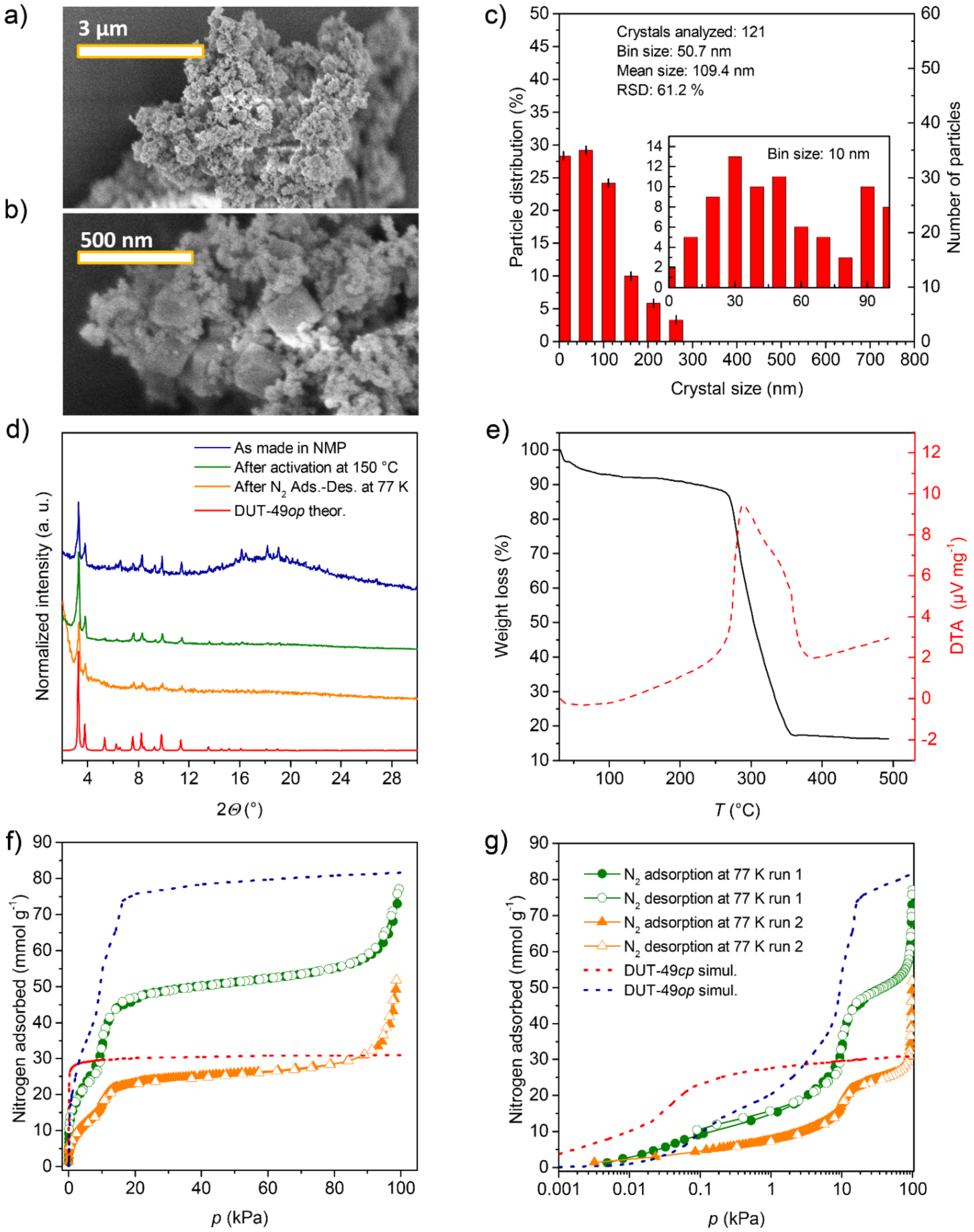

Supplementary Figure 15. Characterization of DUT-49(**15**) (mean size: 110 nm): SEM images (a, b), crystal size distribution derived from SEM images, inlet: magnification of area with smaller crystal sizes and smaller bin size (c), PXRD patters of as made (blue), activated (green), after N_2 adsorption-desorption at 77 K (orange) and theoretical DUT-49*op* (d), TGA (e), and N_2 physisorption at 77 K measured for two consecutive cycles including simulated isotherms for DUT-49*op* (blue dashed line) and DUT-49*cp* (red dashed line) (f); N_2 physisorption isotherms at 77 K as semi-logarithmic plot (g).

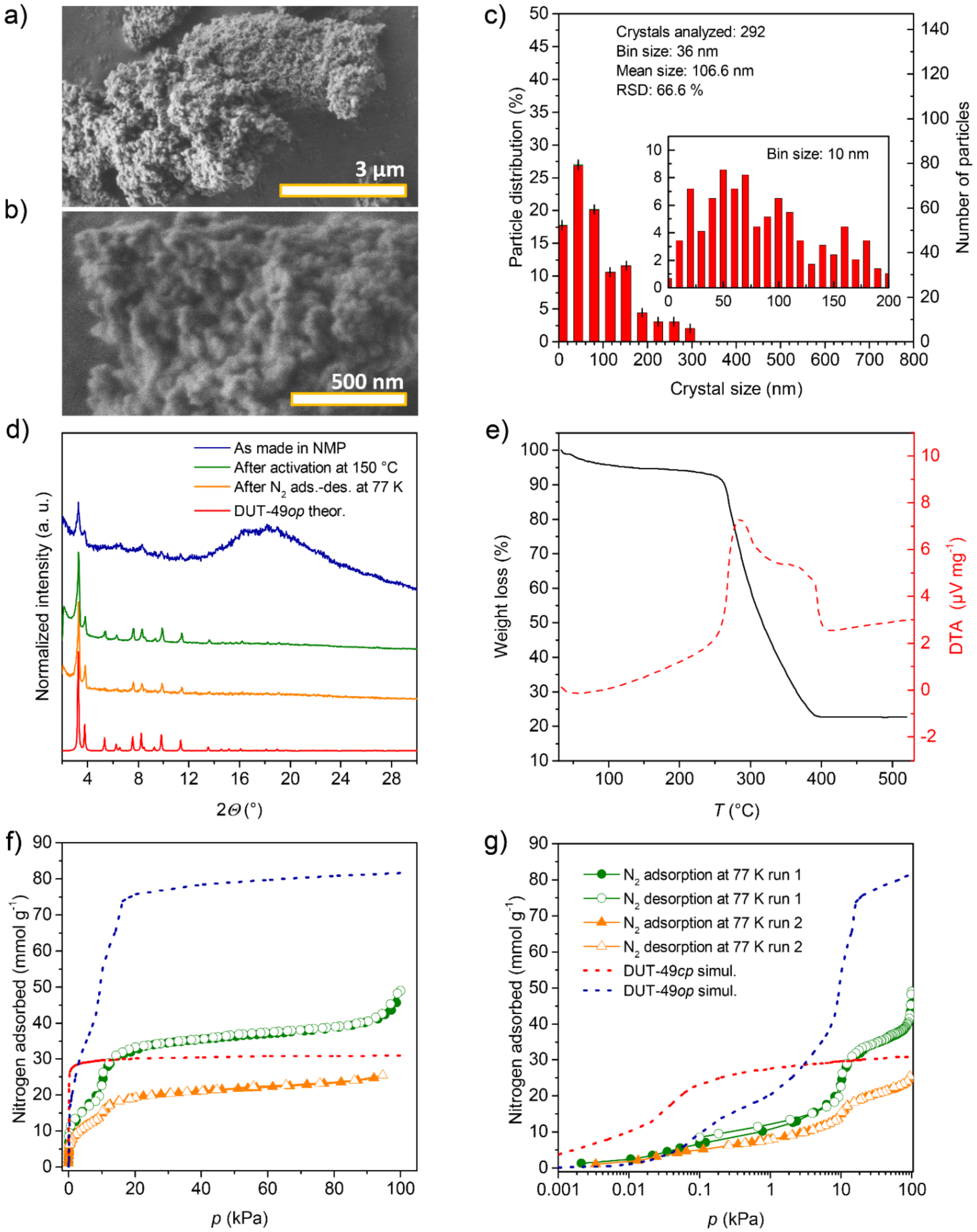

Supplementary Figure 16. Characterization of DUT-49(**16**) (mean size: 107 nm): SEM images (a, b), crystal size distribution derived from SEM images, inlet: magnification of area with smaller crystal sizes and smaller bin size (c), PXRD patters of as made (blue), activated (green), after N_2 adsorption-desorption at 77 K (orange) and theoretical DUT-49*op* (d), TGA (e), and N_2 physisorption at 77 K measured for two consecutive cycles including simulated isotherms for DUT-49*op* (blue dashed line) and DUT-49*cp* (red dashed line) (f); N_2 physisorption isotherms at 77 K as semi-logarithmic plot (g).

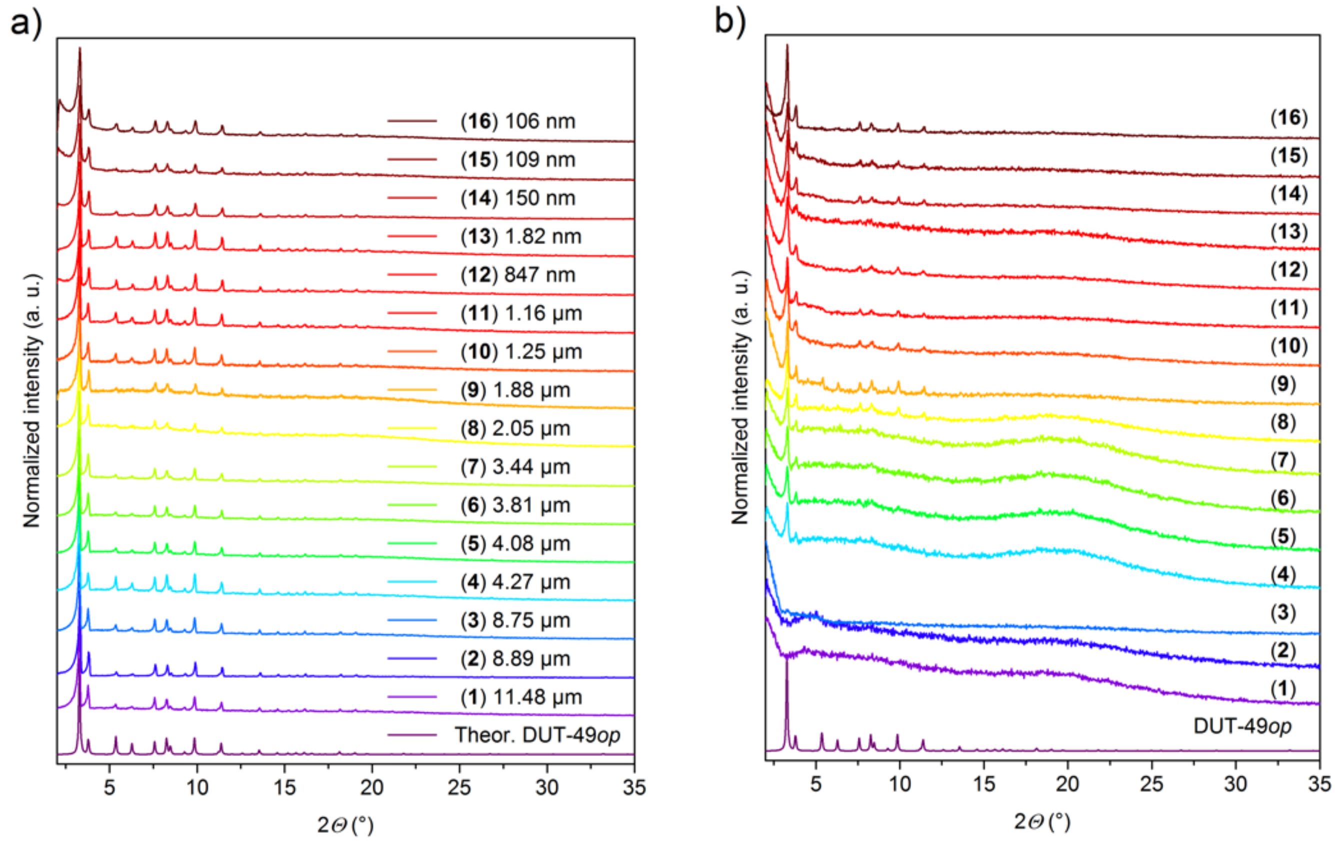

Supplementary Figure 17. PXRD patterns of activated DUT-49 samples (**1**)-(**16**) (supercritical activation followed by thermal activation at 150 °C in dynamic vacuum) (a); and PXRD patterns of DUT-49 samples (**1**)-(**16**) after two cycles of adsorption-desorption of N_2 at 77 K.

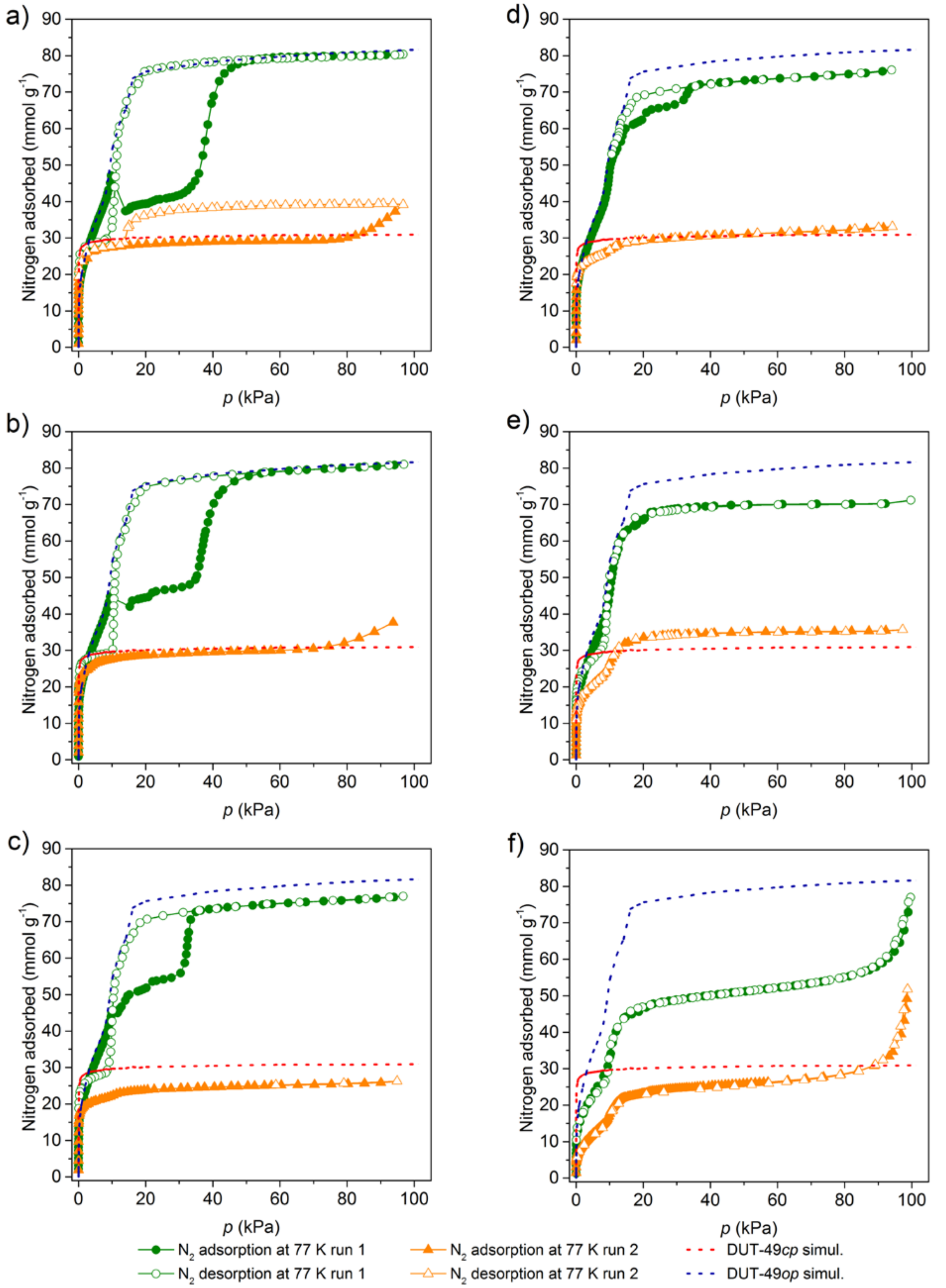

Supplementary Figure 18. Selected N_2 physisorption isotherms at 77 K of DUT-49(**3**) (a), DUT-49(**4**) (b), DUT-49(**10**) (c), DUT-49(**11**) (d), DUT-49(**9**) (e), and DUT-49(**15**) (f). Filled symbols correspond to adsorption, empty symbols to desorption, green circles correspond to first cycle, orange triangles correspond to second cycle. Dashed lines correspond to simulated adsorption isotherms based on the crystal structure of DUT-49*cp* (red) and DUT-49*op* (blue).

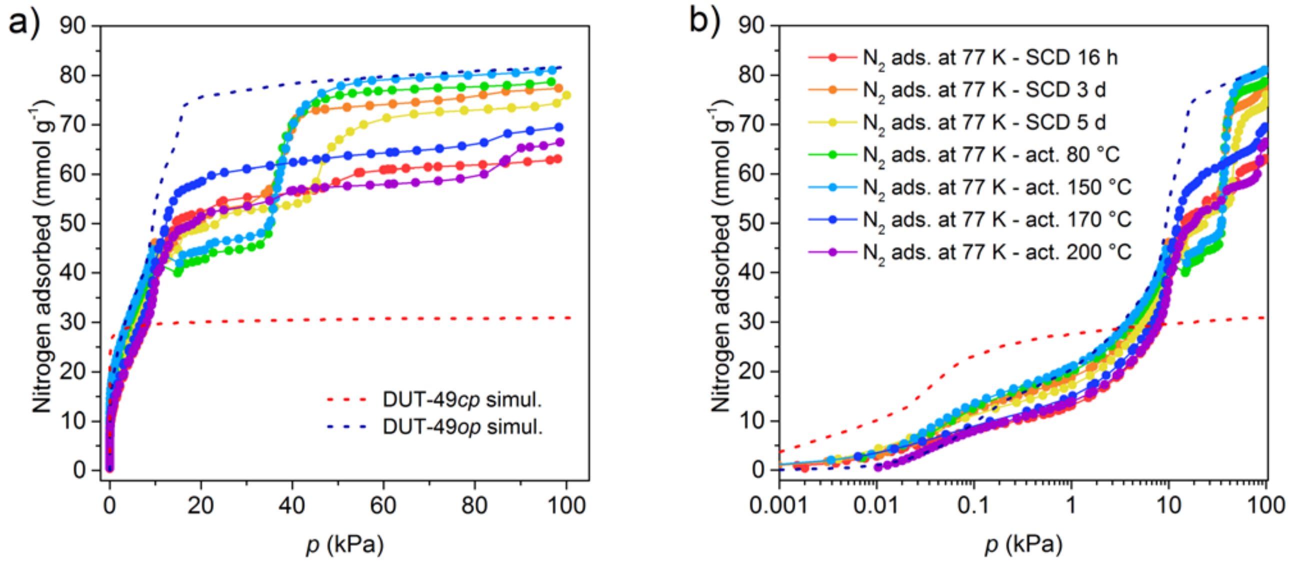

Supplementary Figure 19. N_2 adsorption isotherms at 77 K of DUT-49(**4**) (4.26 µm) obtained for samples activated under different conditions in linear scale (a) and in semi-logarithmic scale (b). Samples were activated using a supercritical activation as described above. For selected samples additional thermal activation which is based on additional treatment in dynamic vacuum at elevated temperatures based on 5 d supercritical dried (SCD) DUT-49 was performed.

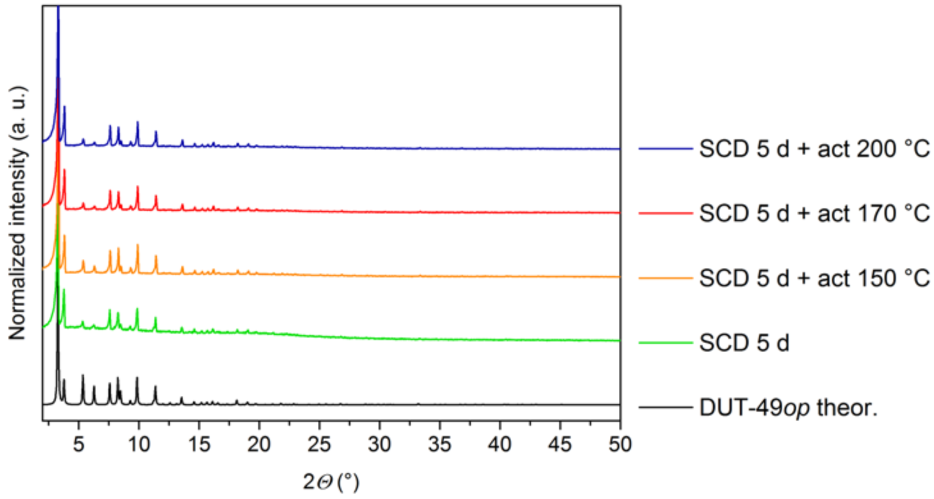

Supplementary Figure 20. PXRD patterns of thermally activated DUT-49(**4**) (4.26 µm) (see Fig. S21 for corresponding N_2 adsorption isotherms at 77 K).

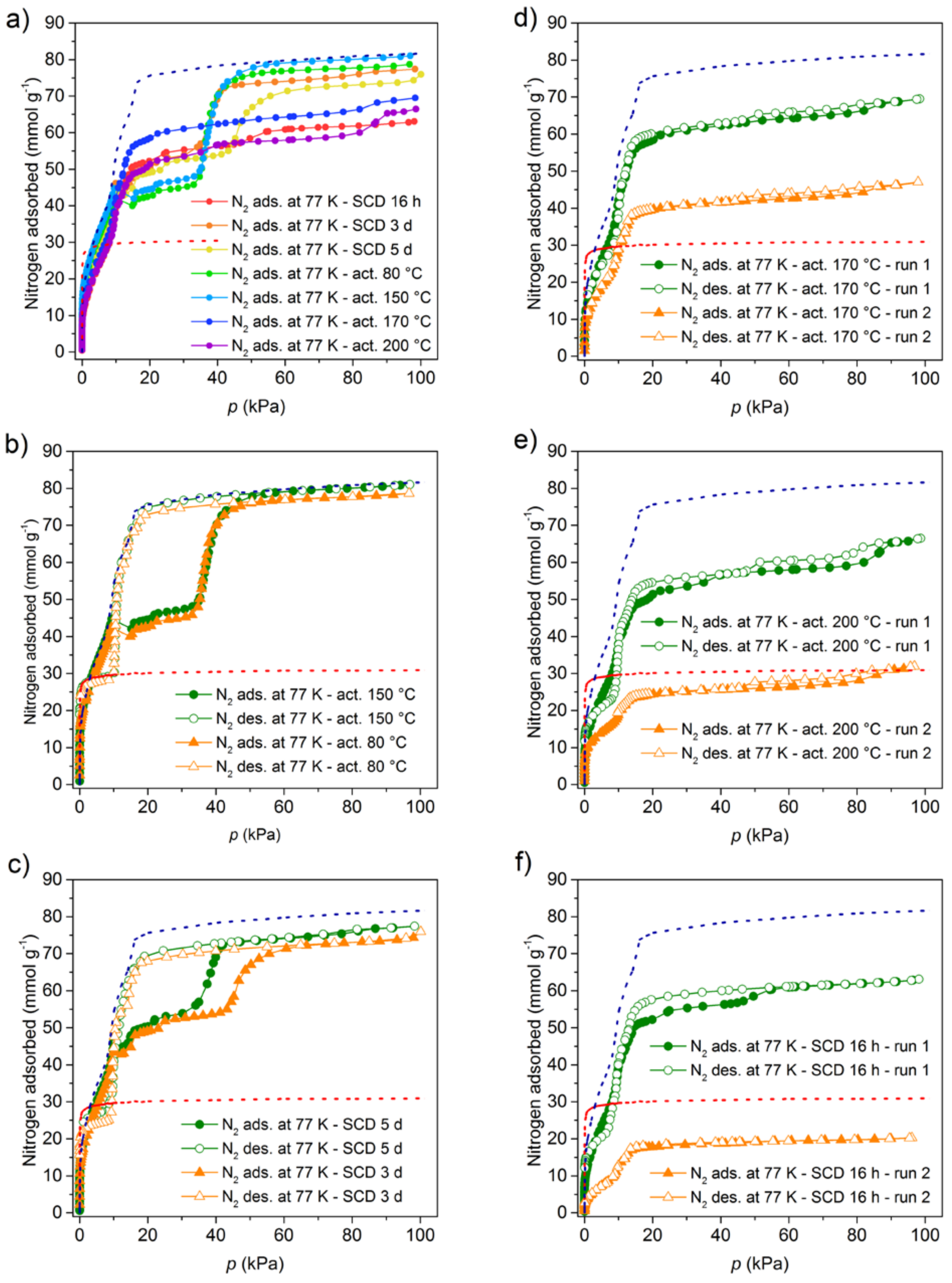

Supplementary Figure 21. N_2 physisorption isotherms at 77 K of DUT-49(**4**) (4.26 µm) activated under different conditions (b-f) and summary of recorded isotherms from Fig S21 (a). Filled symbols correspond to adsorption, empty symbols to desorption. Dashed lines correspond to simulated adsorption isotherms based on the crystal structure of DUT-49*cp* (red) and DUT-49*op* (blue). Thermal activation is based on additional treatment in dynamic vacuum at elevated temperatures after 5 d supercritical drying (SCD) DUT-49.

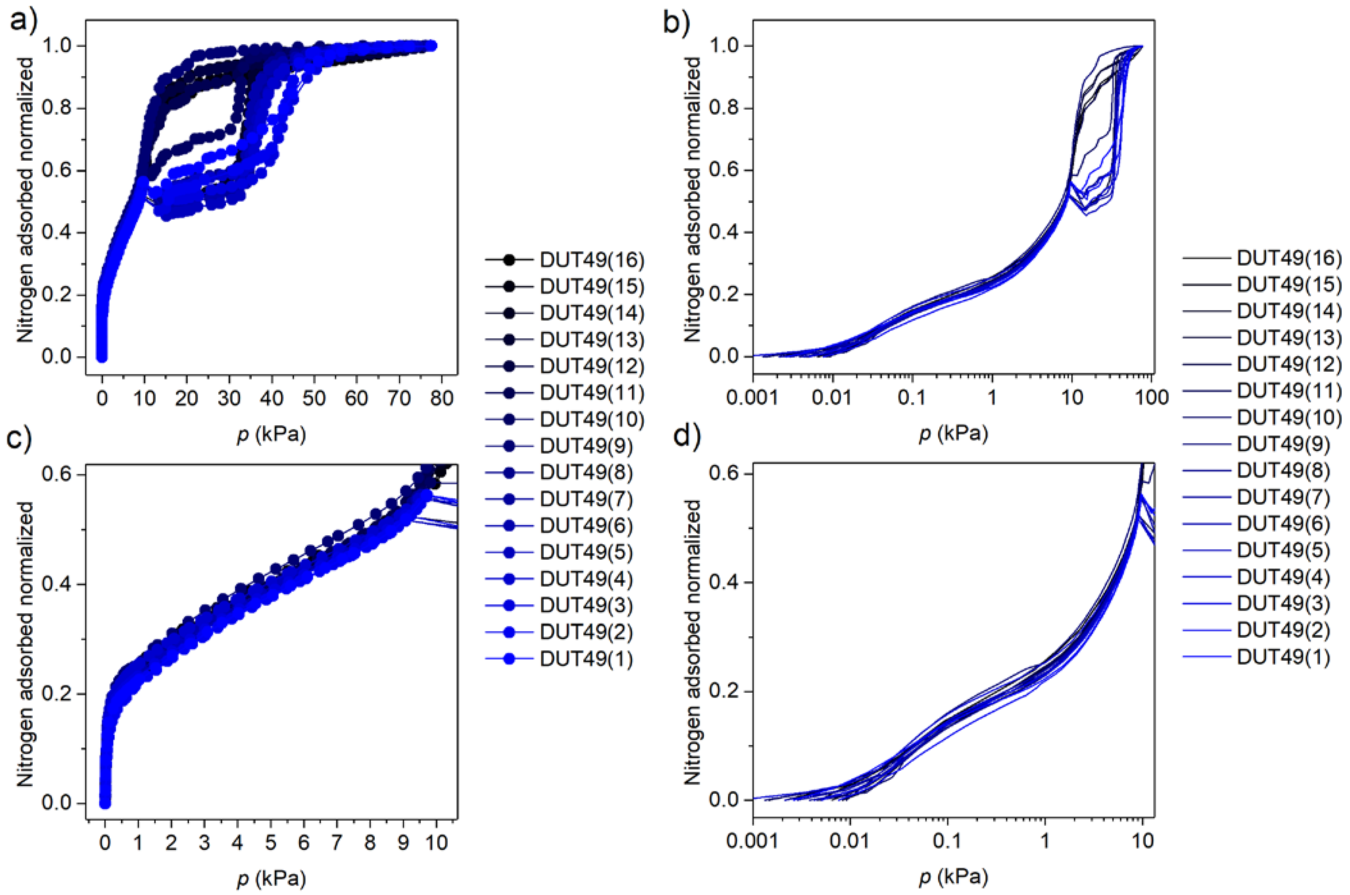

Supplementary Figure 22. Normalized nitrogen adsorption isotherms of DUT-49(1) – DUT-49(16) (based on the uptake at 75 kPa for each isotherm) in regular (a) and semilogarithmic plot (b); c) and d) magnifications of low pressure region of a) and b), respectively.

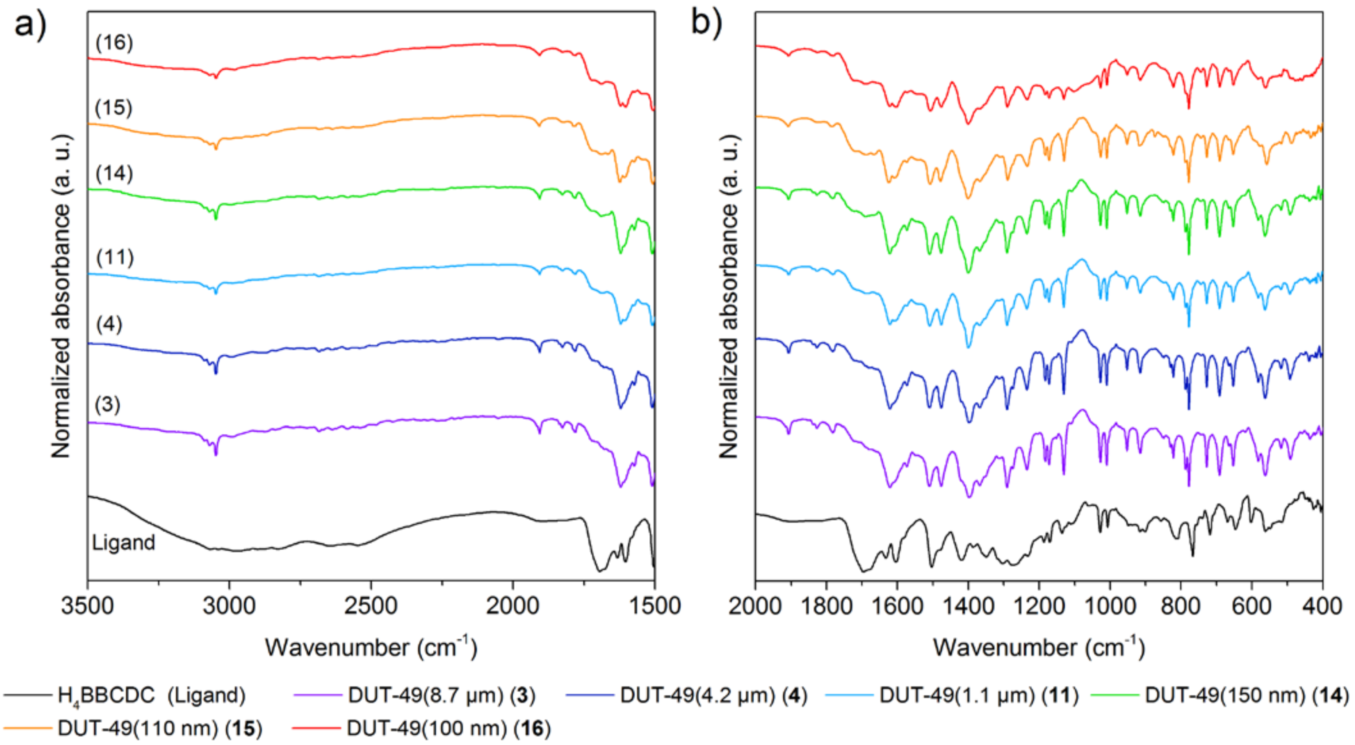

Supplementary Figure 23. DRIFT spectra of H_4BBCDC ligand and activated DUT-49 samples (**3**, **4**, **11**, **14**, **15**, **16**) (a), and enlargement of the spectra fingerprint region (b).

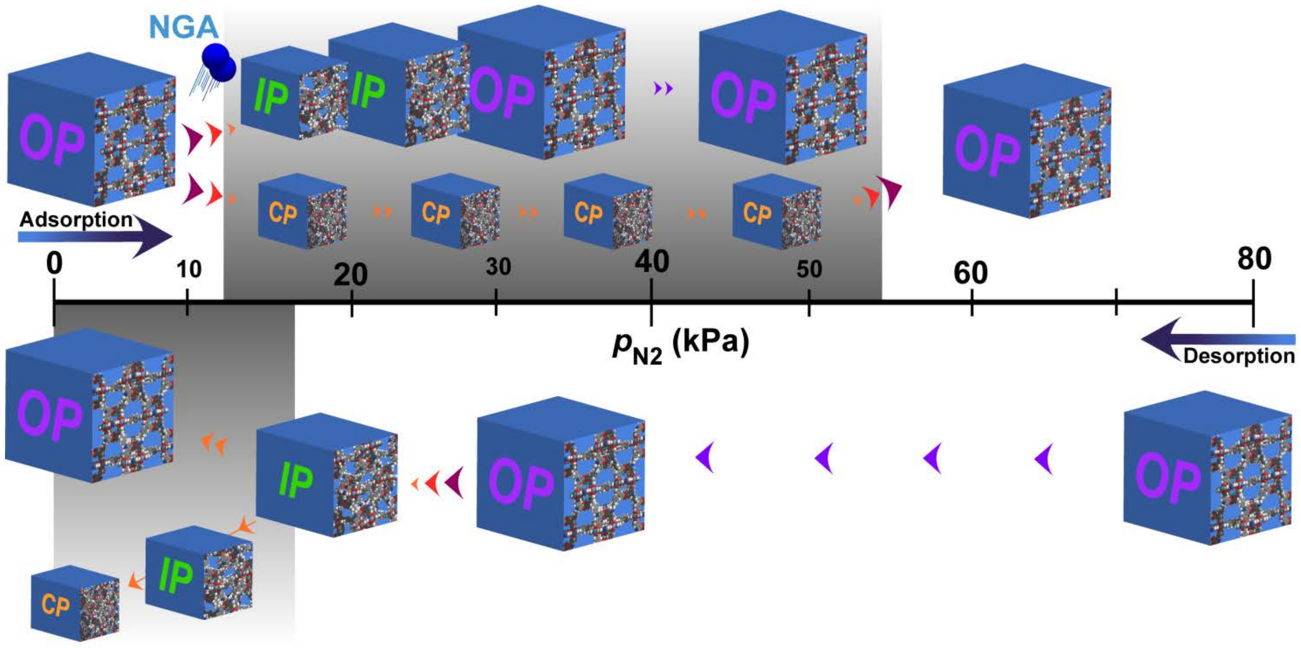

Supplementary Figure 24. Illustration of structural transitions and observed phases during adsorption and desorption of N_2 at 77 in DUT-49(**4**) based on the *in situ* XRD data**.** Pressure regions with phase mixtures of 2 or more phases are highlighted in grey.

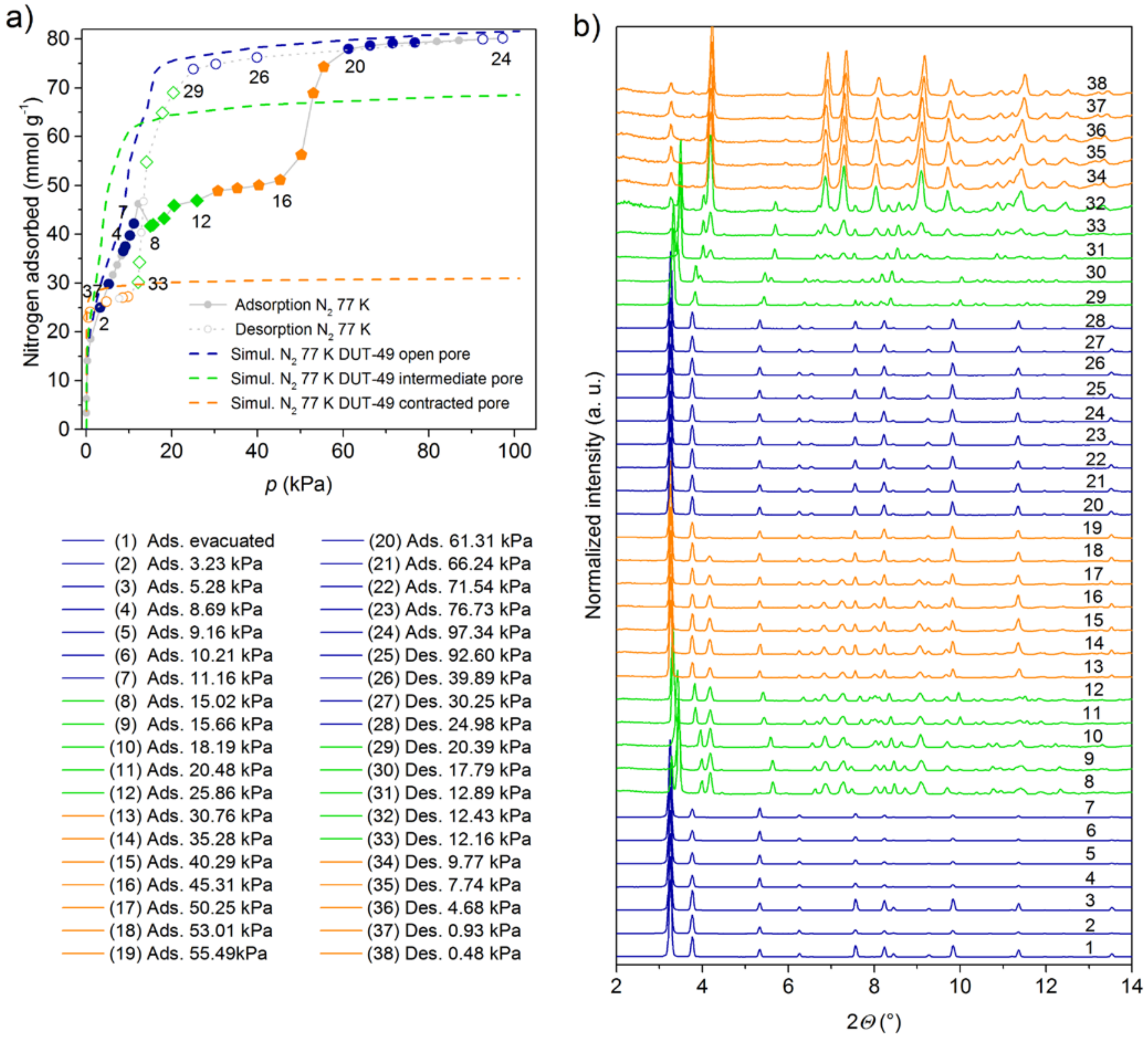

Supplementary Figure 25. *In situ* PXRD in parallel to N_2 physisorption at 77 K of DUT-49(**4**) (4.26 µm): Adsorption-desorption isotherm (a), colored symbols correspond to PXRD patterns in (b), selected points are labeled by numbers. Filled symbols correspond to adsorption, empty symbols to desorption. Dashed lines correspond to simulated adsorption isotherms based on the crystal structure of DUT-49*cp* (orange) DUT-49*ip* (green), and DUT-49*op* (blue).

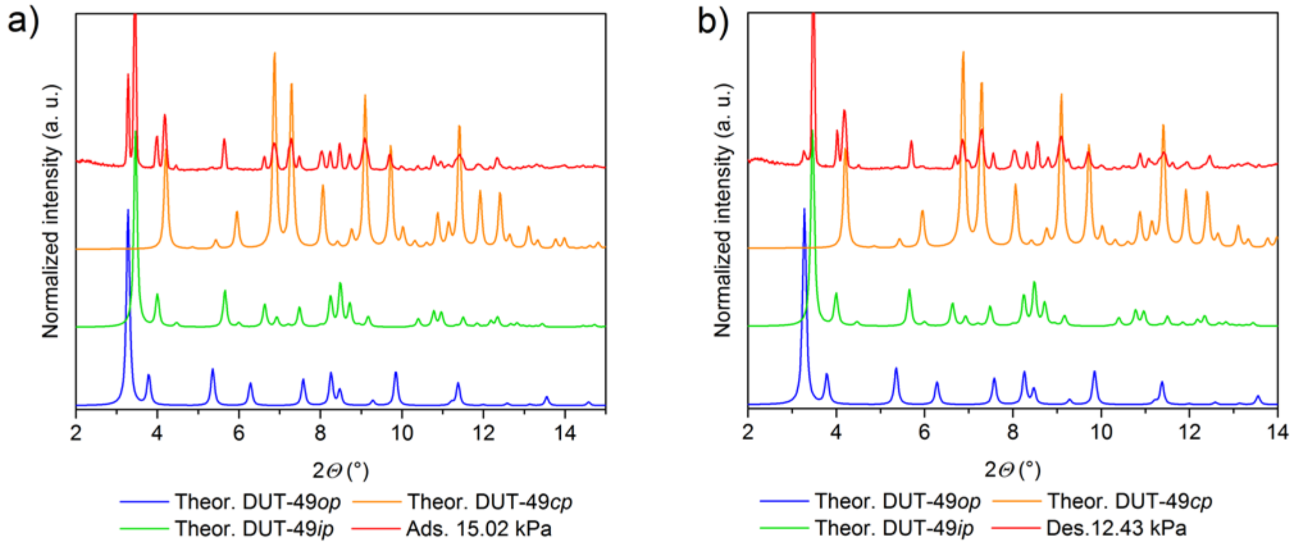

Supplementary Figure 26. Selected PXRD patterns from Supplementary Figure 25 (b) at a N_2 pressure of 15.02 kPa during adsorption (a) and 12.34 kPa during desorption (b) in comparison with theoretical patterns of DUT-49*op* (blue), DUT-49*ip* (green), and DUT-49*cp* (orange) (at 77 K).

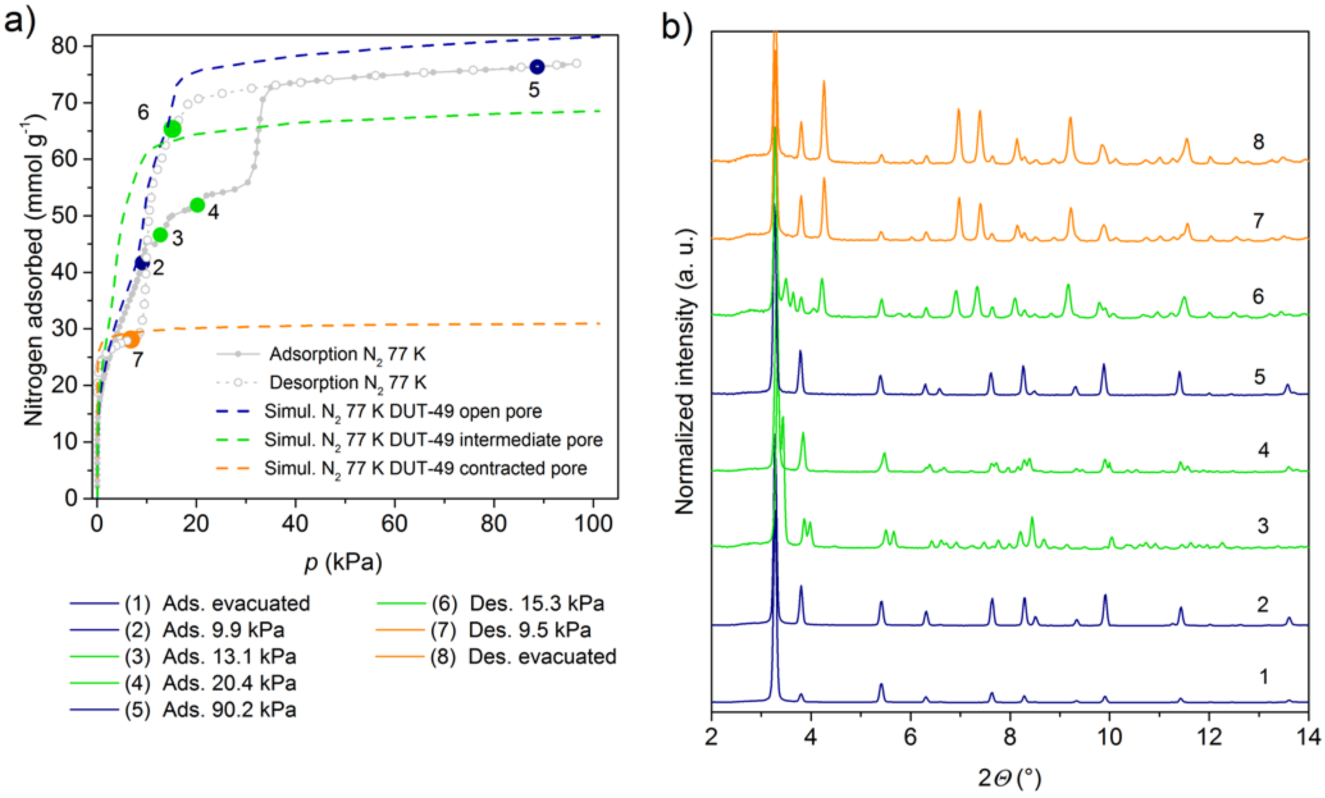

Supplementary Figure 27. *In situ* PXRD in parallel to N_2 physisorption at 77 K of DUT-49(**10**) (1.25 µm): Adsorption-desorption isotherm (a), colored symbols correspond to PXRD patterns in (b), selected points are labeled by numbers. Filled symbols correspond to adsorption, empty symbols to desorption. Dashed lines correspond to simulated adsorption isotherms based on the crystal structure of DUT-49*cp* (orange) DUT-49*ip* (green), and DUT-49*op* (blue).

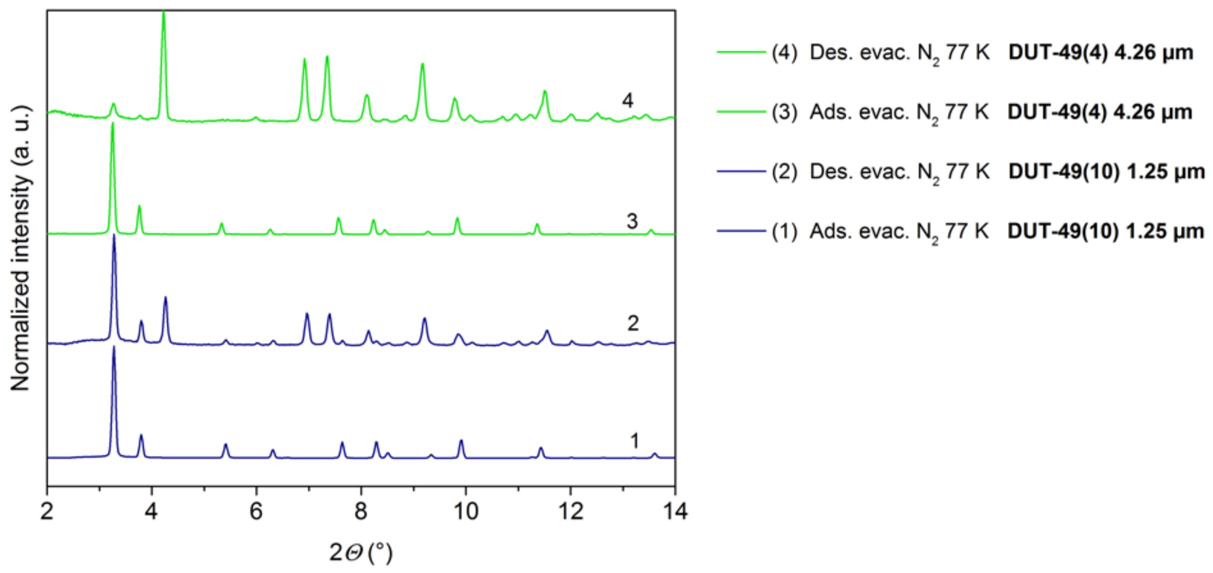

Supplementary Figure 28. Comparison of *in situ* PXRD patterns of DUT-49(**4**) (green) and DUT-49(**10**) (blue), before (bottom) and after (top) N_2 physisorption at 77 K.

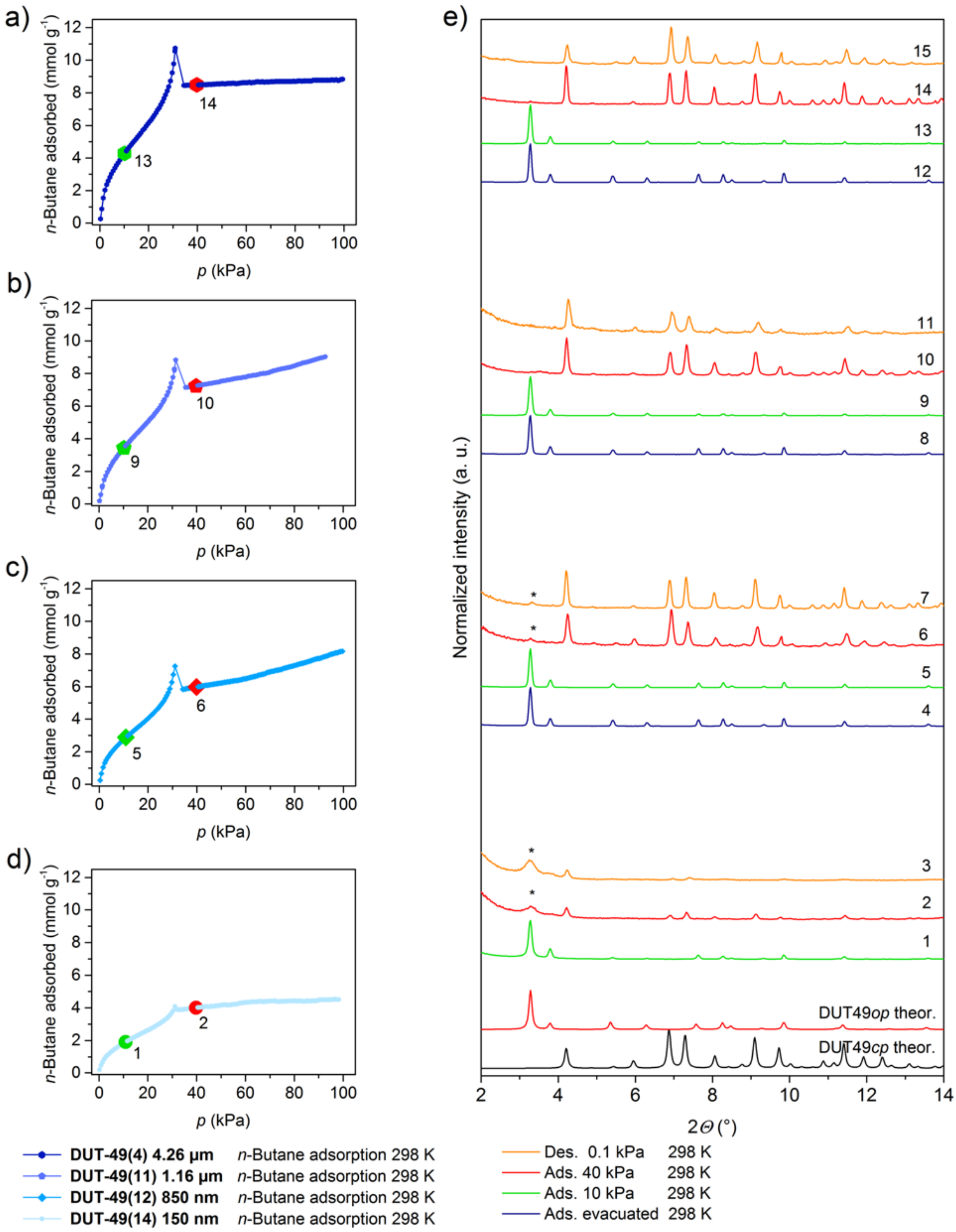

Supplementary Figure 29 *In situ* PXRD (e) collected in parallel to *n*-butane adsorption at 298 K of DUT-49(**4**) (a), DUT-49(**11**) (b), DUT-49(**12**) (c), and DUT-49(**14**) (d). Red and green points mark the pressures at which PXRDs were recorded and correspond to red and green colored PXRD patterns in (e). Blue and orange pattern correspond to evacuated DUT-49 before adsorption and after desorption, respectively. *Remaining reflections of DUT-49*op* in patterns after NGA transition.

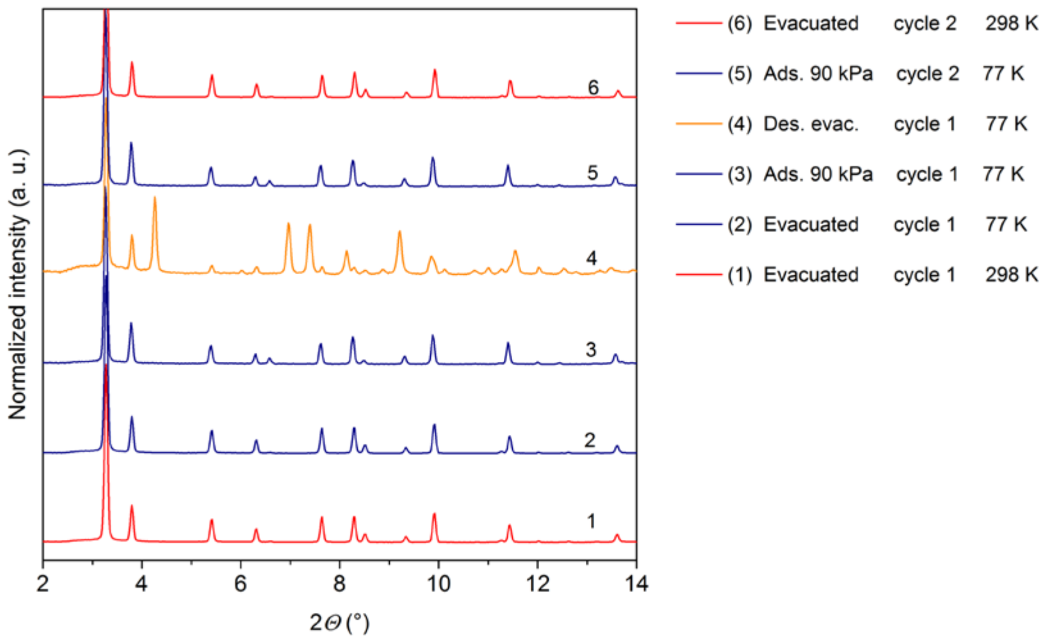

Supplementary Figure 30. N_2 physisorption at 77 K, recycling experiment on DUT-49(**10**) (1.25 µm). Patterns 1-4 correspond to the first cycle from evacuated DUT-49 before adsorption of N_2 at 77 K (2) to evacuated DUT-49 after desorption of N_2 at 77 K (4). Pattern 5 corresponds to the reopened structure after adsorption in the second cycle. Pattern 6 corresponds to the activated material after the temperature has been increased from 77 K to 298 K while maintaining a pressure of 100 kPa N_2 in the measuring cell.

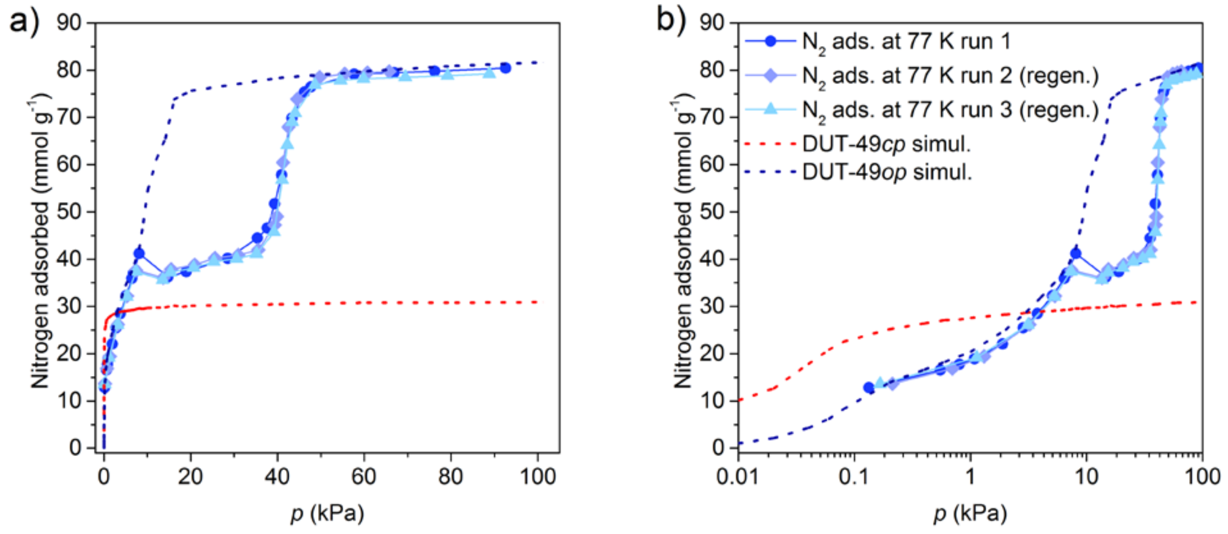

Supplementary Figure 31. Repeated N_2 adsorption isotherms collected at 77 K of DUT-49(**4**) (4.26 µm) in linear (a) and in semi-logarithmic scale (b). Dark blue circles, purple diamond, and light blue triangles correspond to the first, second and third cycle, respectively. Dashed lines correspond to simulated adsorption isotherms based on the crystal structure of DUT-49*cp* (red) and DUT-49*op* (blue). Regeneration (regen.) was performed by warming the sample at 100 kPa N_2 pressure from 77 K to 298 K by removing the sample cell from the bath of liquid N_2.

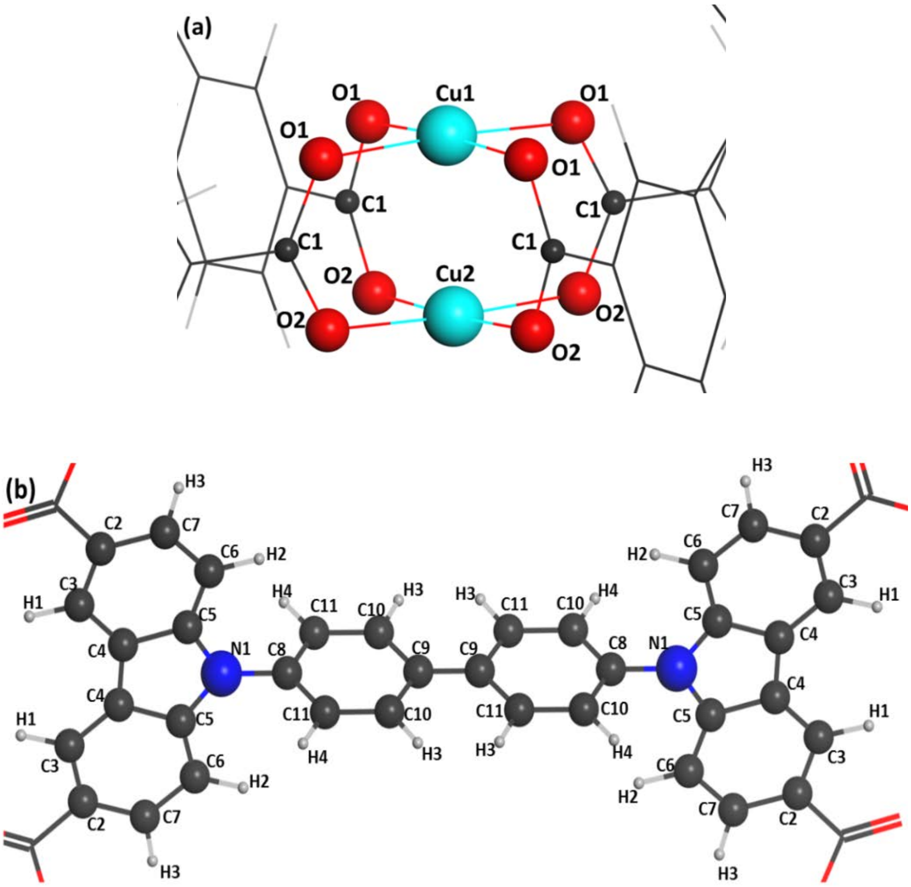

Supplementary Figure 32. Labels of the atoms for the inorganic (a) and organic (b) parts of the DUT-49*op* structure.

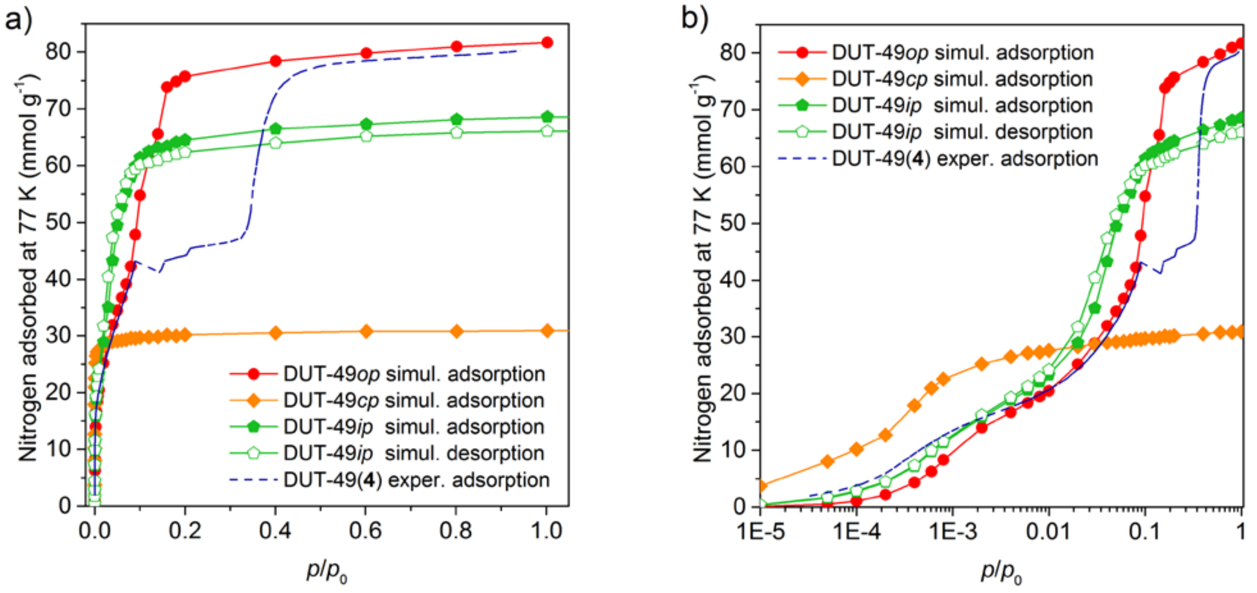

Supplementary Figure 33. Comparison of the pure component simulated and experimental (blue dashed line) adsorption isotherms for N_2 in DUT-49*op* (red circle), DUT-49*ip*-ads (green filled pentagons), DUT-49*cp* (green empty pentagons), and DUT-49*ip*-des (orange diamonds) forms of DUT49 at 77 K (a) and same graph in semi-logarithmic scale (b).

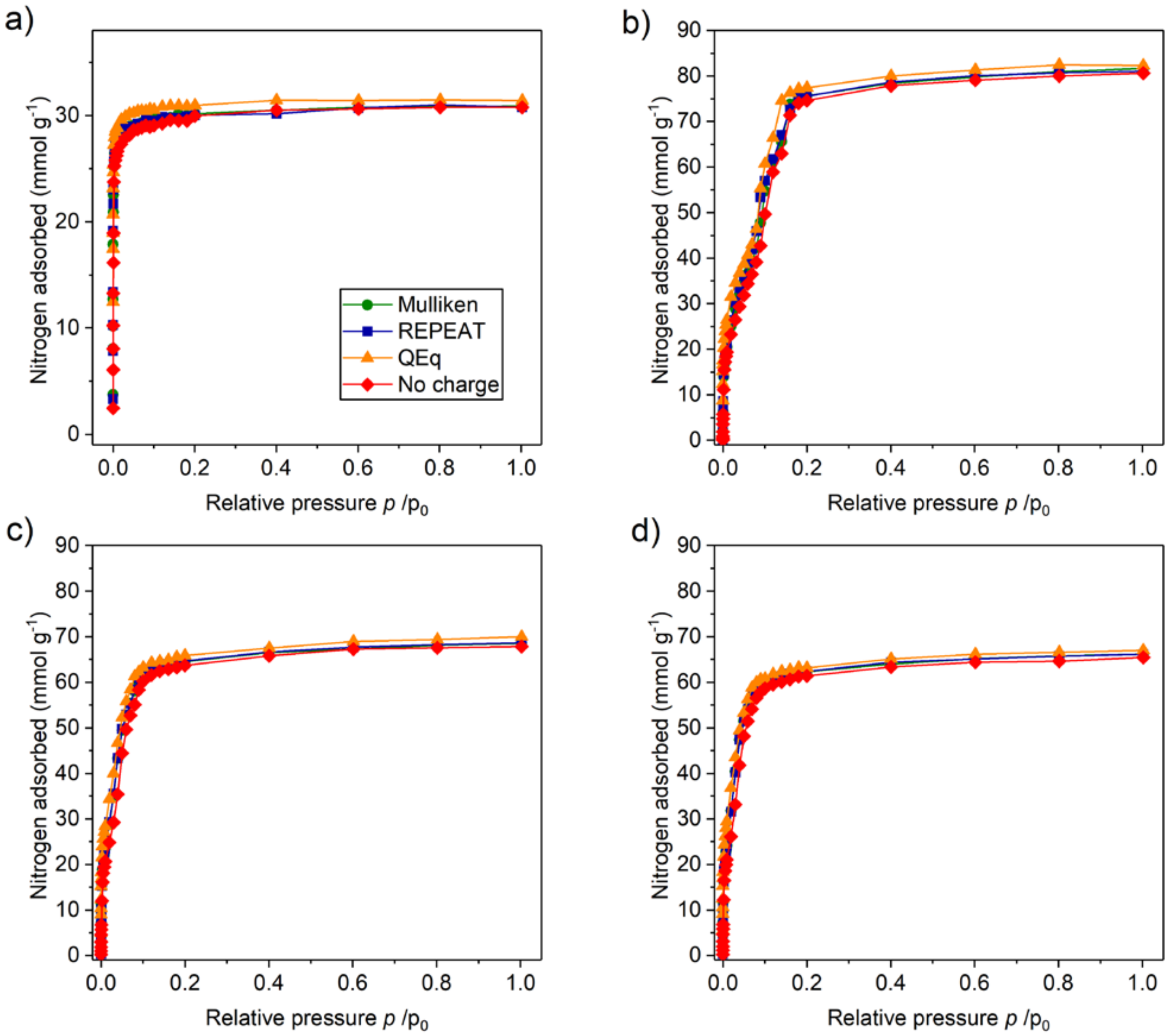

Supplementary Figure 34. Comparison of the simulated adsorption isotherms for N_2 with partial charges Mulliken (green circles), REPEAT (blue squares), QEq (orange triagles), and no partial charges (red diamonds) for DUT49 at 77 K for a) DUT-49*cp*, b) DUT-49*op*, c) DUT-49*ip-ads*, and d) DUT-49*ip-des*.

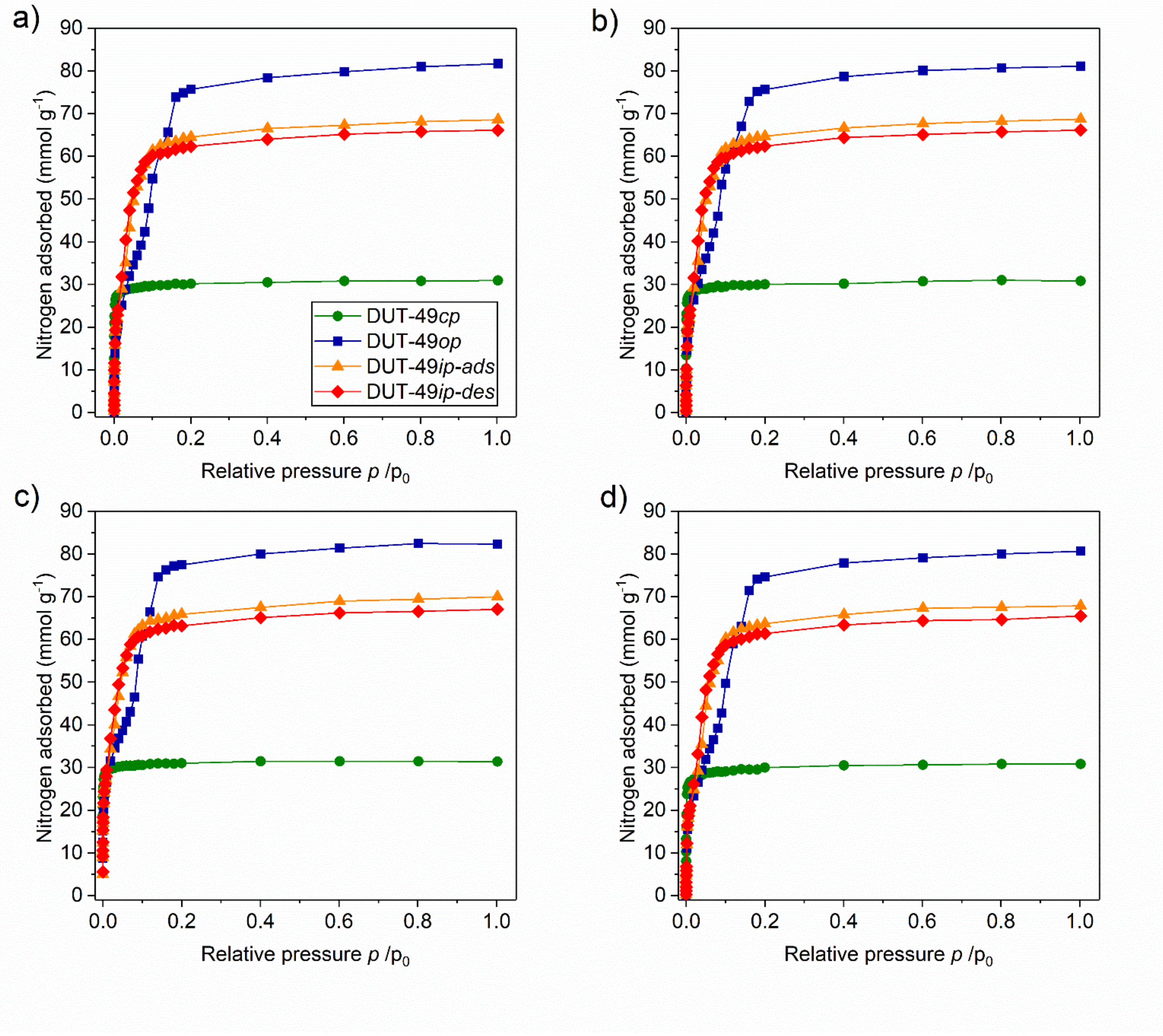

Supplementary Figure 35. Comparison of the simulated adsorption isotherms for N_2 in DUT-49*op* (blue squares), DUT-49*ip*-ads (orange triangles), DUT-49*cp* (green circles), and DUT-49*ip*-des (red diamonds) forms of DUT49 at 77 K for a) Mulliken, b) REPEAT, c) QEd partial charges and d) no partial charges.

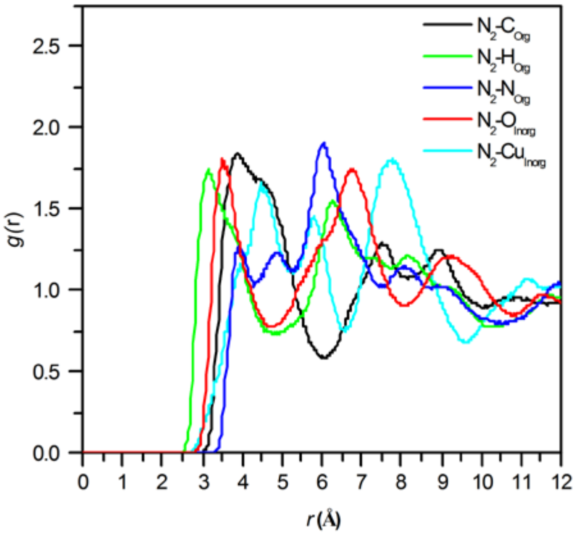

Supplementary Figure 36. Radial distribution functions between N_2 and the carbon, C_{Org}, (black), hydrogen, H_{Org}, (green), nitrogen, N_{Org}, (blue), oxygen, O_{Inorg}, (red), and copper, Cu_{Inorg} (cyan) atoms of the DUT-49*op* at $p/p_0 = 0.1$ and 77 K.

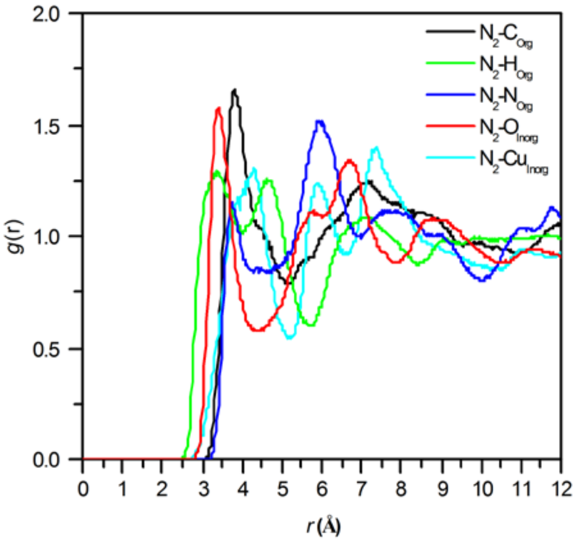

Supplementary Figure 37. Radial distribution functions between N_2 and the carbon, C_{Org}, (black), hydrogen, H_{Org}, (green), nitrogen, N_{Org}, (blue), oxygen, O_{Inorg}, (red), and copper, Cu_{Inorg} (cyan), atoms of the DUT-49*ip*-ads at p/p_0 = 0.11 and 77 K.

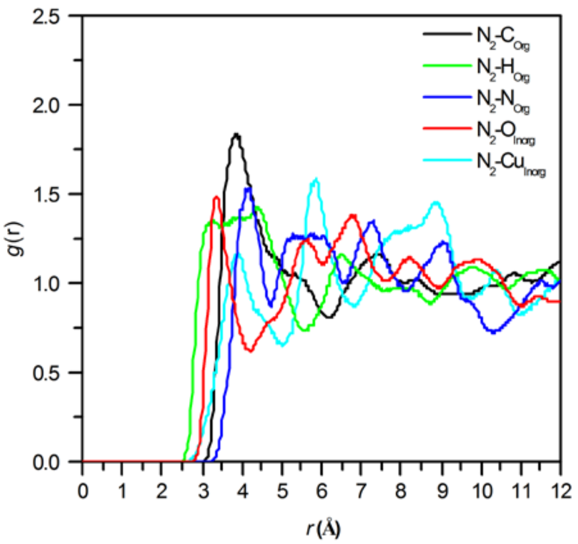

Supplementary Figure 38. Radial distribution functions between N_2 and the carbon, C_{Org}, (black), hydrogen, H_{Org}, (green), nitrogen, N_{Org}, (blue), oxygen, O_{Inorg}, (red), and copper, Cu_{Inorg} (cyan), atoms of the DUT-49*cp* at $p/p_0 = 0.2$ and 77 K.

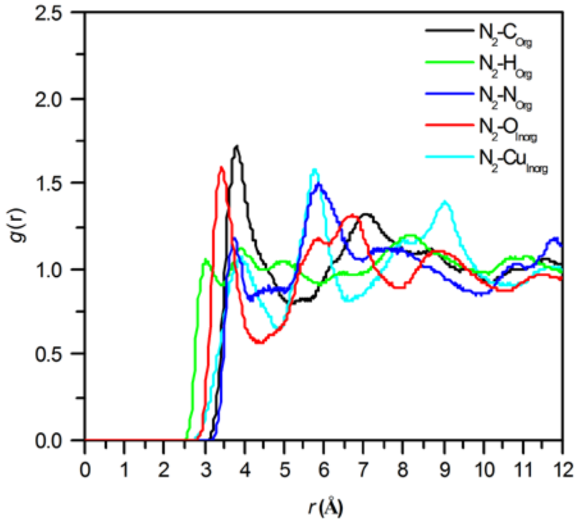

Supplementary Figure 39. Radial distribution functions between N_2 and the carbon, C_{Org}, (black), hydrogen, H_{Org}, (green), nitrogen, N_{Org}, (blue), oxygen, O_{Inorg}, (red), and copper, Cu_{Inorg} (cyan), atoms of the DUT-49*ip*-ads at p/p_0 = 0.15 and 77 K.

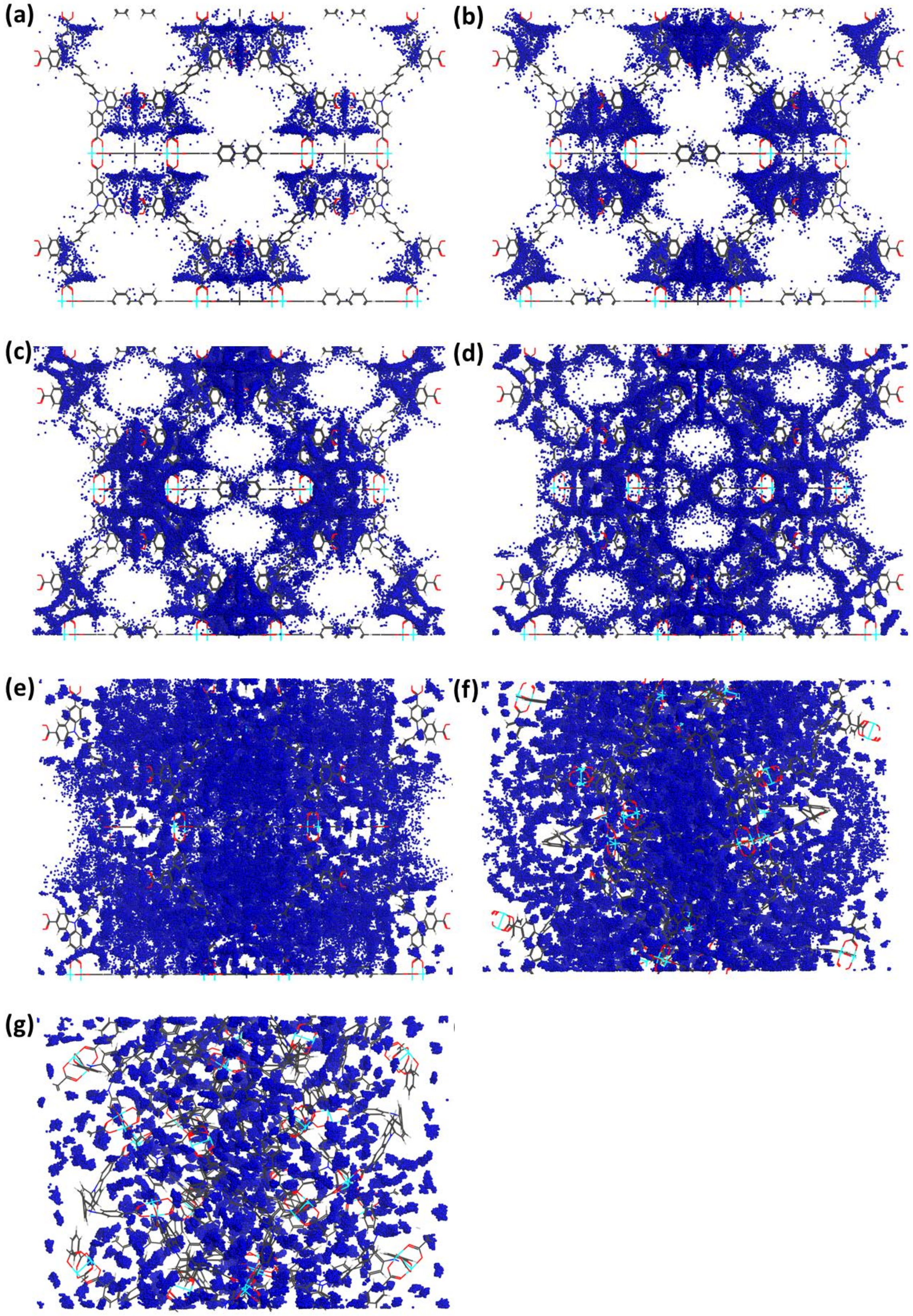

Supplementary Figure 40. Maps of the occupied positions of nitrogen (blue) in 1000 equilibrated frames for a given pressure at 77 K for DUT-49*op* : (a) p/p_0 = 0.0001, (b) p/p_0 = 0.0004, (c) p/p_0 = 0.002, (d) p/p_0 = 0.01 and (e) p/p_o = 0.1, for DUT-49*ip-ads*: p/p_o = 0.11 (f) and DUT-49*cp*: p/p_0 = 0.2 (g) at the transition pressure, color code for the atoms: C (dark grey), N (blue), O (red), Cu (cyan), H (light grey).

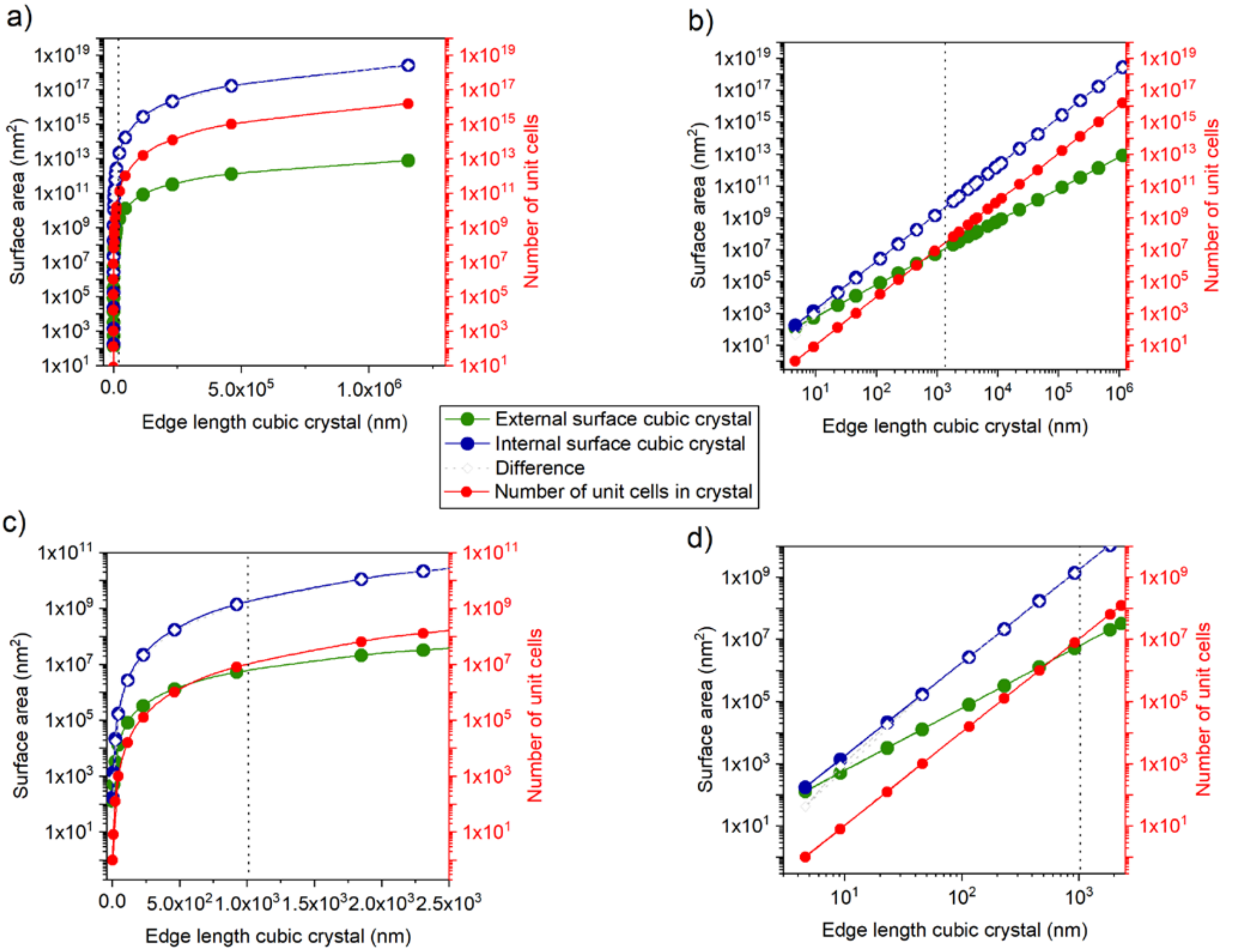

Supplementary Figure 41. Relationship of internal and external surface area and number of unit cells to the crystal edge length in a cubic DUT-49 crystal. External surface area was calculated assuming a flat surface of the cubic crystal. The internal surface per unit cell is 171 nm^2 and was calculated based on the BET surface area of 5476 m^2 g^{-1}. Critical edge length for NGA is marked as dashed line.

Supplementary Table 1. Synthetic conditions for samples synthesized by addition of varying amounts of acetic acid and variation of reaction time.

DUT-49	Mean crystal size (nm)	SD (nm)	RSD (%)	NMP[a] (mL g^{-1})	eq. AcOH[b]	eq. NEt_3[b]	Reaction time (h)	Yield (%)[c]
**1**	11486	±3304	28.7	170	400	0	72	39
**2**	8892	±5307	59.6	172	150	0	72	45
**3**	8750	±2620	29.9	170	300	0	72	43
**4**	4269	±187	43.8	172	150	0	48	44
**5**	4080	±1459	35.7	170	200	0	48	42
**6**	3810	±1804	47.3	170	150	0	48	48
**7**	3443	±1856	53.8	170	100	0	48	46
**8**	2052	±1105	53.8	170	50	0	48	54

[a] mass (g^{-1}) refers to the mass of H_4BBCDC ligand; [b] equivalents based on H_4BBCDC ligand; [c] yield based on H_4BBCDC.

Supplementary Table 2. Synthetic conditions for samples synthesized by variation of ligand amount and reaction time.

DUT-49	Mean crystal size (nm)	SD (nm)	RSD (%)	NMP[b] (mL g^{-1})	eq. AcOH[c]	eq. NEt_3[c]	Reaction time (h)	Yield (%)[d]
**9**[a]	1877	±1812	96.5	179	0	0	36	52
**10**	1255	±1208	96.2	252	0	0	72	55
**11**	1163	±968	83.1	162	0	0	36	54
**12**	848	±507	59.8	152	0	0	36	63

[a] Sample DUT-49(**9**) was synthesized according to reference [1] with longer reaction time of 36 h instead of 24 h; [b] mass (g^{-1}) refers to the mass of H_4BBCDC ligand; [c] equivalents based on H_4BBCDC ligand; [d] yield based on H_4BBCDC.

Supplementary Table 3. Synthetic conditions for samples synthesized by addition of varying NEt_3 equivalents.

DUT-49	Mean crystal size (nm)	RSD (nm)	RSD (%)	NMP[a] (mL g^{-1})	eq. AcOH[b]	eq. NEt_3[b]	Reaction time (h)	Yield (%)[c]
**13**	1828	±853	46.6	152	0	0	16	56
**14**	150	±80	53.5	152	0	0.1	8	57
**15**	109	±67	61.1	152	0	1	8	67
**16**	107	±71	66.6	152	0	4	8	61

[a] mass (g^{-1}) refers to the mass of H_4BBCDC ligand; [b] equivalents based on H_4BBCDC ligand; [c] yield based on H_4BBCDC.

Supplementary Table 4. NGA parameters derived from the N_2 adsorption isotherms at 77 K on samples DUT-49(**1**) – (**16**).

DUT-49	Mean crystal size (nm)	Isotherm	Gas	T (K)	$\|\Delta n_{NGA}\|$ (mmol g^{-1})[a]	Δp (kPa)[a],[b]
1	11486	Supplementary Figure 1	N_2	77	7.842	4.44
2	8892	Supplementary Figure 2	N_2	77	4.33	5.87
3	8750	Supplementary Figure 3	N_2	77	9.66	4.25
4	4269	Supplementary Figure 4	N_2	77	2.73	5.83
5	4080	Supplementary Figure 5	N_2	77	5.04	6.26
6	3811	Supplementary Figure 6	N_2	77	6.98	5.07
7	3444	Supplementary Figure 7	N_2	77	3.86	6.17
8	2052	Supplementary Figure 8	N_2	77	4.09	3.91
9	1877	Supplementary Figure 9	N_2	77	0	0
10	1255	Supplementary Figure 10	N_2	77	0.22	1.85
11	1163	Supplementary Figure 11	N_2	77	0	0
12	848	Supplementary Figure 12	N_2	77	0	0
13	1828	Supplementary Figure 13	N_2	77	3.38	5.89
14	150	Supplementary Figure 14	N_2	77	0	0
15	109	Supplementary Figure 15	N_2	77	0	0
16	107	Supplementary Figure 16	N_2	77	0	0
Ref 1	n. a.	Ref. 1	N_2	77	0	0
Ref 2	n. a.	Ref. 2	N_2	77	0	0

[a] Derived from adsorption isotherm; [b] Δp is the step-change on the pressure axis resulting from the difference in pressure before and after NGA.

Supplementary Table 5. Elemental analyses of various DUT-49 samples.

DUT-49	Mean crystal size (nm)	RSD (%)	NMP (mL g^{-1})	eq. AcOH[a]	eq. NEt_3[a]	Reaction time (h)	N (%)	C (%)	H (%)
calculated[a]							3.57	61.3	2.57
3	8750	29.9	170	300	0	72	3.74	59.93	2.626
4	4269	43.8	172	150	0	48	3.64	59.83	2.557
10	1255	96.2	256	0	0	72	3.83	60.12	2.842
11	1163	83.1	160	0	0	36	3.77	59.85	2.726
14	150	53.5	152	0	0.1	8	3.72	60.87	2.453
15	109	61.1	152	0	1	8	3.78	60.92	2.723
16	107	66.6	152	0	4	8	3.65	56.89	2.842

[a] Equivalents based on H_4BBCDC ligand; [b] calculated based on the solvent free single crystal structure of DUT-49.[2]

Supplementary Table 6. Data obtained from LEBAIL refinement of PXRD patterns in **Supplementary Figure 25 (b)** collected during adsorption of N_2 at 77 K in DUT-49(**4**) (4.26 µm).

		Phase A - DUT-49*op*		Phase B - DUT-49*ip*		Phase C - DUT-49*cp*			
p (kPa)	Pattern Nr. in Supplementary Figure 25	Space group	Lattice parameter *a* (Å)	Space group	Lattice parameter *a* (Å)	Space group	Lattice parameter *a* (Å)	R_p (%)	R_{wp} (%)
0.00	1	$Fm\bar{3}m$	46.6393(3)					7.91	11.70
3.23	2	$Fm\bar{3}m$	46.6643(6)					8.32	11.70
5.29	3	$Fm\bar{3}m$	46.6396(4)					8.12	11.90
8.69	4	$Fm\bar{3}m$	46.6596(6)					6.64	9.62
9.16	5	$Fm\bar{3}m$	46.6588(6)					6.52	9.52
10.22	6	$Fm\bar{3}m$	46.6572(6)					6.57	9.56
11.16	7	$Fm\bar{3}m$	46.6511(6)					7.65	10.60
15.02	8	$Fm\bar{3}m$	46.6214(37)	$Pa\bar{3}$	44.1594(11)	$Pa\bar{3}$	36.3478(14)	4.24	6.43
15.66	9			$Pa\bar{3}$	44.2231(6)	$Pa\bar{3}$	36.3446(8)	3.48	5.31
18.19	10			$Pa\bar{3}$	44.5754(7)	$Pa\bar{3}$	36.3711(7)	4.10	5.97
20.48	11			$Pa\bar{3}$	45.8698(6)	$Pa\bar{3}$	36.4009(8)	3.72	5.23
25.86	12			$Pa\bar{3}$	46.0507(6)	$Pa\bar{3}$	36.4243(8)	3.89	5.59
30.76	13	$Fm\bar{3}m$	46.6693(5)			$Pa\bar{3}$	36.4314(7)	4.74	6.83
35.28	14	$Fm\bar{3}m$	46.6910(4)			$Pa\bar{3}$	36.4535(7)	3.84	5.66
40.29	15	$Fm\bar{3}m$	46.7025(5)			$Pa\bar{3}$	36.4697(8)	4.54	6.84
45.31	16	$Fm\bar{3}m$	46.7020(5)			$Pa\bar{3}$	36.4790(8)	3.82	5.50
50.25	17	$Fm\bar{3}m$	46.7061(4)			$Pa\bar{3}$	36.4925(9)	3.60	5.33
53.01	18	$Fm\bar{3}m$	46.7076(4)			$Pa\bar{3}$	36.4911(13)	3.86	5.65
55.49	19	$Fm\bar{3}m$	46.7058(4)			$Pa\bar{3}$	36.4998(26)	3.52	5.34
61.31	20	$Fm\bar{3}m$	46.7106(4)			$Pa\bar{3}$	36.5042(159)	4.31	6.33
66.24	21	$Fm\bar{3}m$	46.7119(4)					3.94	5.65
71.54	22	$Fm\bar{3}m$	46.7136(4)					4.20	6.21
76.73	23	$Fm\bar{3}m$	46.7181(4)					4.33	6.50
97.34	24	$Fm\bar{3}m$	46.7317(4)					4.22	6.19

Supplementary Table 7. Data obtained from LeBail refinement of PXRD patterns collected during desorption of N_2 at 77 K in DUT-49(**4**) (4.26 µm) (Supplementary Figure 25(b)).

		Phase A - DUT-49*op*		Phase B - DUT-49*ip*		Phase C - DUT-49*cp*			
p (kPa)	Pattern Nr. in Supplementary Figure 25	Space group	Lattice parameter *a* (Å)	Space group	Lattice parameter *a* (Å)	Space group	Lattice parameter *a* (Å)	R_p (%)	R_{wp} (%)
92.60	25	$Fm\bar{3}m$	46.7295(3)					4.25	6.00
39.89	26	$Fm\bar{3}m$	46.6913(4)					4.49	6.71
30.25	27	$Fm\bar{3}m$	46.6768(4)					4.45	6.48
24.98	28	$Fm\bar{3}m$	46.6698(4)					4.63	6.47
20.39	29	$Fm\bar{3}m$	46.6689(11)	$Pa\bar{3}$	45.8596(5)			3.76	5.43
17.80	30			$Pa\bar{3}$	45.7114(6) / 44.4861(7)			3.32	4.83
12.89	31	$Fm\bar{3}m$	46.3930(78)	$Pa\bar{3}$	43.8030(2)	$Pa\bar{3}$	36.3377(12)	3.56	5.51
12.43	32	$Fm\bar{3}m$	46.5319(71)	$Pa\bar{3}$	43.7075(6)	$Pa\bar{3}$	36.3215(7)	3.59	5.44
12.17	33	$Fm\bar{3}m$	46.5541(25)	$Pa\bar{3}$	43.6491(13)	$Pa\bar{3}$	36.3343(7)	3.96	6.08
9.77	34	$Fm\bar{3}m$	46.5603(84)			$Pa\bar{3}$	36.3009(7)	5.06	8.38
7.74	35	$Fm\bar{3}m$	46.5420(100)			$Pa\bar{3}$	36.2713(7)	5.17	8.54
4.68	36	$Fm\bar{3}m$	46.4569(138)			$Pa\bar{3}$	36.2213(7)	5.30	8.74
0.93	37	$Fm\bar{3}m$	46.3676(204)			$Pa\bar{3}$	36.0705(6)	5.44	8.56
0.48	38	$Fm\bar{3}m$	46.3776(62)			$Pa\bar{3}$	36.0204(5)	5.00	7.93

Supplementary Table 8. Data obtained from LeBail refinement of PXRD patterns collected during adsorption and desorption (grey background) of N_2 at 77 K in DUT-49(**10**) (1.25 µm) (Supplementary Figure 27 (b)).

		Phase A - DUT-49*op*		Phase B - DUT-49*ip*		Phase C - DUT-49*cp*			
p (kPa)	Pattern Nr. in Supplementary Figure 27	Space group	Lattice parameter a (Å)	Space group	Lattice parameter a (Å)	Space group	Lattice parameter a (Å)	Rp (%)	Rwp (%)
0	1	$Fm\bar{3}m$	46.6020(7)					12.0	17.4
9.9	2	$Fm\bar{3}m$	46.5963(8)					7.89	12.7
13.1	3			$Pa\bar{3}$	45.8166(8) / 44.5255(7)			4.40	6.88
20.4	4			$Pa\bar{3}$	46.0990(15)			9.51	16.6
90.2	5	$Fm\bar{3}m$	46.7255(5)					6.91	9.92
15.3 (Des)	6			$Pa\bar{3}$	45.7613(10) / 44.2315(10)			5.38	8.55
9.5 (Des)	7			$Pa\bar{3}$	46.5745(7)	$Pa\bar{3}$	36.0453(4)	4.43	6.50
0 (Des)	8			$Pa\bar{3}$	46.5606(8)	$Pa\bar{3}$	36.0646(4)	4.01	5.80

Supplementary Table 9. NGA related properties extracted from the *n*-butane adsorption isotherms and kinetic profiles at 298 K.

DUT-49	Mean crystal size (nm)	Gas	T (K)	$\|\Delta n\|$ mmol g^{-1} [a]	Δp (kPa) [a]	$\|\Delta p(NGA)\|$ (kPa)	$\Delta p(NGA)$ (kPa g^{-1}) [b]
**4**	4269	*n*-butane	298	2.28	3.54	2.75	298.6
**11**	1163	*n*-butane	298	1.69	4.04	2.31	281.3
**12**	848	*n*-butane	298	1.42	3.18	2.09	183.3
**14**	150	*n*-butane	298	0.16	0.90	0.39	46.5
**Ref. [1]**	n. a.	*n*-butane	298	1.44	8.11	2.58	208

[a] Extracted from *n*-butane adsorption isotherm; [b] extracted from the kinetic profile during NGA; [c] $V_{sample\ cell}$ was constant and was ca. 21.2 cm^3 for adsorption of *n*-butane.

Supplementary Table 10. Mulliken partial charges for all atoms of DUT-49.

Atomic types	C1	C2	C3	C4	C5	C6	C7	C8
Charge (e)	0.573	-0.018	-0.146	0.017	0.269	-0.136	-0.142	0.127
Atomic types	C9	C10	C11	Cu1	Cu2	H1	H2	H3
Charge (e)	0.088	-0.099	-0.162	0.465	0.473	0.154	0.128	0.144
Atomic types	H4	H5	N1	O1	O2			
Charge (e)	0.124	0.147	-0.612	-0.448	-0.441			

Supplementary Table 11. REPEAT partial charges for all atoms of DUT-49.

Atomic types	C1	C2	C3	C4	C5	C6	C7	C8
Charge (e)	0.666	-0.132	-0.088	-0.088	0.275	-0.311	-0.092	0.493
Atomic types	C9	C10	C11	Cu1	Cu2	H1	H2	H3
Charge (e)	0.507	-0.301	-0.453	0.977	0.949	0.161	0.152	0.152
Atomic types	H4	H5	N1	O1	O2			
Charge (e)	0.191	0.173	-0.252	-0.592	-0.572			

Supplementary Table 12. QEq partial charges for all atoms of DUT-49.

Atomic types	C1	C2	C3	C4	C5	C6	C7	C8
Charge (e)	0.331	0.009	-0.162	0.014	0.162	-0.134	-0.163	0.158
Atomic types	C9	C10	C11	Cu1	Cu2	H1	H2	H3
Charge (e)	0.032	-0.133	0.108	2.675	2.887	0.071	0.108	0.093
Atomic types	H4	H5	N1	O1	O2			
Charge (e)	0.087	0.094	-0.405	-0.775	-0.775			

Supplementary Table 13. LJ potential parameters for the atoms of DUT-49.

Atomic type	LJ potential	
	σ (Å)	ε/k_B (K)
C	3.473	47.857
H	2.846	7.649
N	3.661	38.975
O	3.118	30.195
Cu	3.295	2.518

Supplementary Table 14. LJ potential parameters and partial charges for nitrogen and *n*-butane.

Atomic type	σ (Å)	ε/k_B (K)	q (e)
N_2_N	3.310	36.000	-0.4820
N_2_COM	0.00	0.000	0.9640
CH_3_sp^3	3.750	98.000	0.0
CH_2_sp^2	3.950	46.000	0.0

Supplementary Table 15. Enthalpy values for N_2 at 77 K and zero coverage in DUT-49.

DUT-49	$\left\|\Delta_{ads}\dot{h}_{\theta=0}(N_2)\right\|$ / kJ mol^{-1}
	N_2
DUT-49*op*	-10.0
DUT-49*ip*-ads	-11.4
DUT-49*cp*	-13.0
DUT-49*ip*-des	-11.7

Supplementary Discussion

Rationalization of synthesis conditions:

From the conducted variations of the DUT-49 synthesis we derive the following relationships: Crystal size can be increased by using a lower concentration of the ligand, using higher concentrations of acetic acid, extending the reaction time and stirring the reaction mixture. Smaller crystals can be obtained by concentrating the reaction mixture, using no acetic acid, using a base such as Et_3N and using shorter reaction times. The standard deviation which describes the dispersity of the size distribution is the lowest for samples synthesized using high concentrations of modulator (acetic acid or Et_3N) or for stirred solutions without the addition of modulator. Samples which were synthesized with highly diluted reaction mixtures (sample DUT-49(**9**), (**10**)) show a broad crystal size distribution.

To obtain a very narrow distribution of crystal sizes the nucleation should be controlled with a low concentration of nuclei and a continuous growth of the seeds with a low or negligible additional nucleation after the initial seed formation. In the samples with high SD (especially sample DUT-49(**9**), (**10**) with high dilution of the ligand) we assume that nucleation takes place continuously throughout the reaction time, consequently producing smaller crystals by renewed nucleation while previously formed seeds continue to grow.

Calculation of internal and external surface area

The ratio of external to internal surface area in DUT-49 was estimated as shown in Supplementary Fig. 41. The difference between internal and external surface grows significantly with crystal size (edge length). As a result, external surface distortion, pore blocking, and amorphous surface layers may play a larger role in smaller crystals and cause a reduced total uptake. In this work, samples with reduced crystal size below 2 µm show an increased clustering and intergrowth of the crystals while samples with larger crystals seem to form individual separated particles (also see SEM images Supplementary Fig. 1-16). Such factors may impact the total uptake and pore volume of smaller crystals stronger than in larger crystals. Another aspect is the reduced density of the adsorbed phase in the outer layer causing an apparent reduction of surface area by crystal downsizing as shown for microporous MOFs like ZIF-8. This effect and its particle size dependence in mesoporous MOFs needs further investigations in future.

However, a hint for such a decreased adsorbate density in the outer layer may be indicated in the adsorption isotherms for samples DUT-49(**14**)-(**16**). These isotherms do not reach a plateau and show a tendency towards IUPAC type II behaviour with a positive slope indicating that mesopores on the outer layer are filled at higher relative pressure than those located deeper inside the volume of DUT-49. A similar sloping is also detected for the smaller particles in *n*-butane isotherms.

Refinement of PXRD patterns

From our previous report on methane adsorption on DUT-49[1] at 111 K it is known that the material undergoes a discontinuous structural transition during methane adsorption. The transition involves the reduction of the symmetry from $Fm\bar{3}m$ (*op* phase) to $Pa\bar{3}$ (*cp* phase, cell parameter smaller than 35 Å) and a colossal unit cell volume change. Desorption of the methane is accompanied by two phase transitions: a) continuous phase transition from *op* phase to *ip* phase; b) discontinuous transition from *ip* ($Pa\bar{3}$) phase to *cp* ($Pa\bar{3}$) phase. Therefore, the corresponding crystal structures were used as starting models for the LeBail profile matching during nitrogen adsorption at 77 K (Supplementary Fig. 25). The refinement shows that PXRD patterns 1 - 7, 21 - 28 and 34 - 38 in Fig. S25 contain pure *op* and *cp* phase, whereas in all other points, phase mixtures of *ip* + *cp* (9 - 12), *cp* + *op* (13 - 20, 34 - 38), *ip* + *op* (29) or even three phases (8, 31 - 33) are present. Corresponding unit cell parameters are given in Supplementary Tab. 6-7. Thus, after reaching the threshold pressure of 12 kPa, the pronounced NGA effect could be observed in the adsorption isotherm. LeBail fit of PXRD pattern 8 during nitrogen adsorption suggests the co-existence of all three phases *cp* + *ip* + *op*, which is different from methane

adsorption at 111 K, where only the *cp* phase is observed. PXRD patterns 9 - 12 represent a two-phase mixture of *ip* and *cp*, whereas the unit cell constants of *ip* continuously increase with increasing pressure and subsequently transform into *op* phase. This also involves an increase of the uptake in the nitrogen adsorption isotherm. PXRD patterns 13 - 20 involve the two-phase mixture of *cp* + *op* phases, whereas intensity of reflections of *cp* phase gradually decrease by approaching the plateau in the isotherm, in which only single *op* phase could be detected. Desorption is characterized by co-existence of the phase mixtures as well. PXRD pattern 29, measured at 20.39 kPa, already involves the *op* + *ip* phase mixture. Further desorption leads to continuous decrease of the unit cell parameters of *ip* phase from a = 45.86 Å (PXRD 29) to a = 43.65 Å (PXRD 33). At lower pressures, only *cp* phase with the impurity of *op* phase exists. The unit cell constants of the *cp* phases vary in a narrow range of 36.34 – 36.02 Å, confirming the thermodynamic stability of this phase at given conditions.

GCMC Simulations

Supplementary Fig. 34 reports the comparison of the simulated N_2-adsorption isotherms at 77 K for the different DUT-49 forms using the three different set of charges (Mulliken, REPEAT and qEq) along with the data obtained when the coulombic interaction is switched off. It is clearly observed that the adsorption of N_2 is primarily governed by the vdw interactions making the choice of the method even more arbitrary as it is evidenced by only a minor change of the adsorption isotherms whatever the set of charges employed.

Supplementary References

1 Krause, S. *et al.* A pressure-amplifying framework material with negative gas adsorption transitions. *Nature* **532**, 348-352, (2016).

2 Stoeck, U., Krause, S., Bon, V., Senkovska, I. & Kaskel, S. A highly porous metal-organic framework, constructed from a cuboctahedral super-molecular building block, with exceptionally high methane uptake. *Chem. Commun.* **48**, 10841-10843, (2012).
